# Diverse oncogenes use common mechanisms to drive growth of major forms of human cancer

**DOI:** 10.1126/sciadv.adt1798

**Published:** 2025-08-20

**Authors:** Otto Kauko, Mikko Turunen, Päivi Pihlajamaa, Antti Häkkinen, Rayner M. L. Queiroz, Mirva Pääkkönen, Sami Ventelä, Massimiliano Gaetani, Susanna L. Lundström, Antonio Murgia, Biswajyoti Sahu, Johannes Routila, Gong-Hong Wei, Heikki Irjala, Julian L. Griffin, Kathryn S. Lilley, Teemu Kivioja, Sampsa Hautaniemi, Jussi Taipale

**Affiliations:** ^1^ Applied Tumor Genomics Program, University of Helsinki, Biomedicum, P.O. Box 63, Haartmaninkatu 8, Helsinki FIN-00014 University of Helsinki, Finland.; ^2^ Department of Biochemistry, University of Cambridge, Cambridge, UK.; ^3^ Turku Bioscience, University of Turku and Åbo Akademi University, Turku, Finland.; ^4^ Department of Systems Biology, Columbia University Irving Medical Center, New York, NY, USA.; ^5^ Research Program in Systems Oncology, University of Helsinki, Biomedicum, P.O. Box 63, Haartmaninkatu 8, Helsinki FIN-00014 University of Helsinki, Finland.; ^6^ Cambridge Centre for Proteomics, University of Cambridge, Cambridge, UK.; ^7^ Department for Otorhinolaryngology–Head and Neck Surgery, University of Turku and Turku University Hospital, Turku, Finland.; ^8^ Chemical Proteomics Core Facility, Division of Physiological Chemistry I, Department of Medical Biochemistry and Biophysics, Karolinska Institutet, Stockholm, Sweden.; ^9^ Chemical Proteomics Unit, SciLifeLab (Science for Life Laboratory), Stockholm, Sweden.; ^10^ Centre for Molecular Medicine Norway, University of Oslo, Oslo, Norway.; ^11^ Disease Networks Research Unit, Faculty of Biochemistry and Molecular Medicine & Biocenter Oulu, University of Oulu, Oulu, Finland.; ^12^ Rowett Institute, University of Aberdeen, Aberdeen, UK.; ^13^ Department of Computer Science, University of Helsinki, P.O. Box 68, Helsinki FIN-00014 University of Helsinki, Finland.; ^14^ Department of Medical Biochemistry and Biophysics, Karolinska Institutet, Stockholm, Sweden.

## Abstract

Mutations in numerous genes contribute to human cancer, with different oncogenic lesions prevalent in different cancer types. However, the malignant phenotype is simple, characterized by unrestricted cell growth, invasion, and often metastasis. One possible hypothesis explaining this dichotomy is that cancer genes regulate common targets, which then function as master regulators of essential cancer phenotypes. To identify mechanisms that drive the most fundamental feature shared by all tumors—unrestricted cell proliferation—we used a multiomic approach, which identified translation and ribosome biogenesis as common targets of major oncogenic pathways across cancer types. Proteomic analysis of tumors and functional studies of cell cultures established nucleolar and coiled-body phosphoprotein 1 as a key node, whose convergent regulation, both transcriptionally and posttranslationally, is critical for tumor cell proliferation. Our results indicate that lineage-specific oncogenic pathways regulate the same set of targets for growth control, revealing key downstream nodes that could be targeted for therapy or chemoprevention.

## Teaser

Oncogenic mutations affecting transcription and phosphorylation converge on regulation of ribosome biogenesis.

## INTRODUCTION

Mutations in more than 500 genes have been causally linked to cancer (*1*, *2*). Several genes exist that are commonly mutated in many different cancer types, such as *p53*, *TERT*, *ATM*, and *CDKN2A* (*3*, *4*). These genes are implicated in processes such as maintaining DNA integrity and progression through cell cycle checkpoints. However, most known cancer genes exhibit a high degree of tissue specificity. Notably, genes encoding proteins involved in growth signal transduction, including members of the Wnt, Hedgehog, and tyrosine kinase/RAS/phosphatidylinositol 3-kinase (PI3K) signaling pathways, exhibit some of the highest observed mutation frequencies in select cancers while not being mutated at observable rates in others (*3*, *5*, *6*).

Growth signal transduction promotes the expression of genes needed for cell proliferation. Some signal transduction pathways, such as Wnt and Hedgehog, activate specific transcription factors (TFs) with well-known target genes (*7*–*9*). Activation of tyrosine kinase/RAS/PI3K signaling can also regulate activity of specific TFs (*10*–*12*), but most of their downstream targets have an unknown function (*13*). It is thus unclear whether transcriptional regulation is the main shared outcome of phosphorylation signaling or whether its growth-promoting effect is also transduced more directly via effects on protein activity levels, e.g., via up-regulation of metabolism and translation (*14*).

**Fig. 1.**
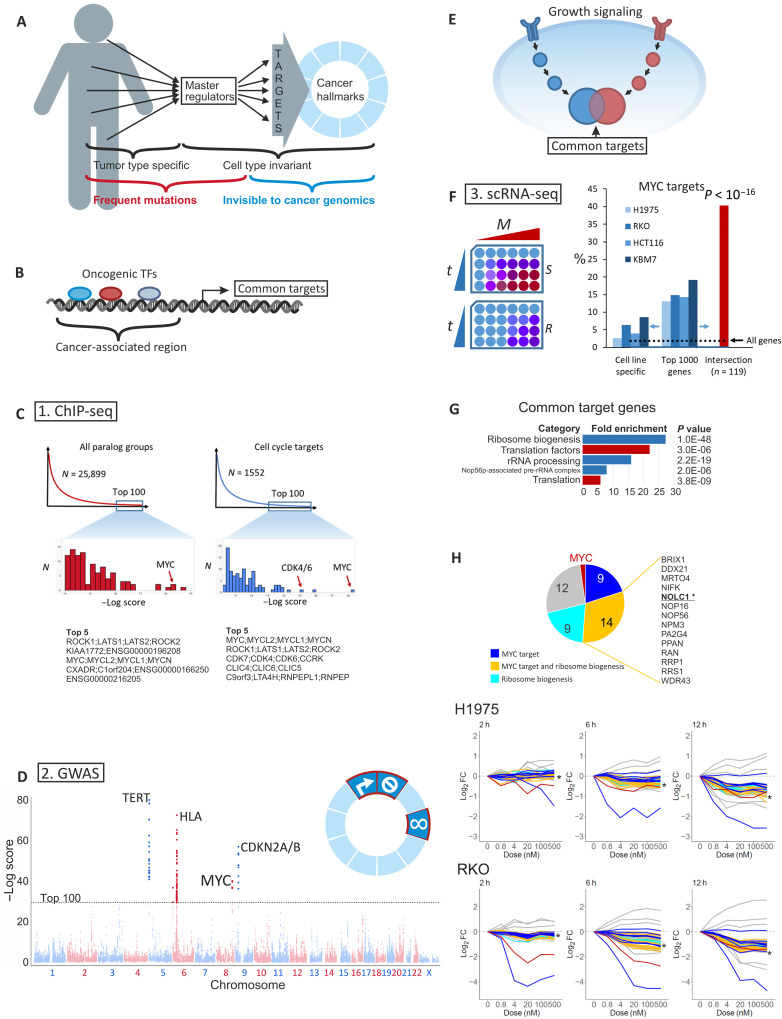
Transcriptional regulation of cell growth. (**A**) Hypothesized model: Diverse pathways and oncogenes drive cell cycle by targeting a limited set of downstream genes, which function as master regulators of the hallmarks of cancer. (**B**) Oncogenic TFs may share functions by binding to the enhancers of shared downstream genes. (**C**) Shared targets of oncogenic TFs were identified by ChIP-seq. Left: Top-scoring shared targets based on the number and heights of ChIP-seq peaks and their proximity to transcription start sites. Right: Top-scoring targets with a conserved role in cell cycle regulation (*25*, *26*). (**D**) Cancer-associated single-nucleotide polymorphisms (SNPs) in GWAS catalog. Top 100 highest scoring SNPs are above the dashed line. Highest scoring SNPs are from four loci corresponding to *TERT*, HLA antigens, *MYC*, and *CDKN2A/B*. (**E**) Phosphorylation pathways may exhibit convergent functions if the downstream kinases phosphorylate (functionally) overlapping sites. (**F**) Cells were treated with varying concentrations of drugs (0 to 500 nM), and scRNA-seq was performed at 2-, 6-, 12-, and 24-hour time points. Genes were ranked for each cell line pair based on the explanatory power of drug treatment in the sensitive cells in a multiple regression model. Top genes exhibited significant overlap between cell lines and were highly enriched in MYC targets (see Materials and Methods). (**G**) g:Profiler (*102*) enrichment analysis of the common targets using Benjamini-Hochberg multiple hypothesis correction method. Ribosome biogenesis, Gene Ontology (GO):0042254; translation factors, WP:WP107; ribosomal RNA (rRNA) processing, REAC:R-HSA-72312; Nop56p-associated pre-rRNA complex, CORUM:3055; translation, GO:0006412. (**H**) Expression of common target genes as a function of drug concentration at 2-, 6-, and 12-hour (h) time points. Intersection of the top 500 genes is shown for sensitive cell lines. Red, MYC; dark blue, MYC targets; cyan, ribosome biogenesis (GO:0042254); yellow, MYC target involved in ribosome biogenesis; *, NOLC1. FC, fold change. The data in (F) to (H) were generated from NCI-H1975, RKO, HCT116, and KBM7/HAP1 cells (table S2).

Established genetic methods have inherent limitations in their capacity to systematically identify all targets for mechanism-based cancer therapy and prevention. Somatic cancer genetics identifies genes that cause cancer, not proteins whose inactivation specifically kills cancer cells. In addition, while a somatic mutagenesis “screen” may be saturating with respect to individual genes and amino acids, it lacks the power to detect combinations of more than ~10 mutations. This can lead to a failure to detect large protein complexes and metabolic pathways whose tumor growth-promoting activity requires increase in levels or activity of multiple subunits or pathway components. In such a case, each member of a pathway or protein complex can still be individually necessary for the oncogenic activity and thus represent a potentially druggable target. Given that genetic approaches have innate limitations in detecting potential drug targets, we hypothesize that a variety of presently unknown mechanisms exist whose activity could be targeted to treat or prevent cancer (Fig. 1A).

Here, we have studied both transcriptional and posttranslational mechanisms in relevant cancer types covering half of population-level cancer mortality to identify common outcomes of oncogenic signaling. The TFs analyzed have all been previously shown to be critical for the formation of the tumors and are involved in a substantial fraction of all cases of the respective forms of cancer (table S1). Similarly, the analyzed phosphorylation signaling pathways are frequently activated in their respective cancers (table S1). The selected cancer types are associated with diverse tissues and represent a substantial fraction of all cancer morbidity, which supports the generalizability of the findings [see (*4*, *7*, *15*–*22*)].

We report here that, of 10 hallmarks of cancer (*23*), the three that are related to cell proliferation (sustaining proliferative signaling, evading growth suppressors, and enabling replicative immortality) are the most prominent shared downstream targets of oncogenic signaling in these cancers. No genes were identified that specifically affect two other central cancer hallmarks, invasion and metastasis, suggesting that these processes may depend on tumor type–specific mechanisms. Our study provides further insight into regulation of ribosome biogenesis and translation by rigorous analysis of fitness effects of specific gene regulatory and phosphorylation events. We further demonstrate that the optimal activation of these processes requires that same proteins are both up-regulated (by MYC) and phosphorylated (by downstream kinases of the Ras/tyrosine kinase pathway), revealing a mechanistic explanation for the well-established but poorly understood phenomenon of oncogene cooperativity. Last, we show that the identified mechanisms display specificity to proliferating tumor cells compared to other cells with high protein synthesis capacity.

## RESULTS

### Oncogenic TFs from major forms of human cancer regulate MYC

Targeting oncogenic drivers in any particular tumor results in a complex cell type– and drug-specific response and causes a broad spectrum of changes in gene expression and posttranslational modifications. Therefore, we reasoned that generating a large number of datasets from different tumor types and focusing on the common features across multiple of them would enable us to focus on a more limited set of downstream mechanisms that are critical for cell proliferation regardless of tumor type. To ensure that all data were comparable, we generated all the primary input datasets to this study in-house, using consistent methodology and equipment.

We first set out to identify common transcriptional targets of oncogenic TFs. We reasoned that convergence of oncogenic transcriptional mechanisms would most likely occur via enhancer elements (Fig. 1B). Key master regulators would contain multiple enhancers, and the tissue specificity of oncogenes would be explained by the fact that a given oncogenic TF activates a particular enhancer by collaborating with tissue-specific factors (*24*).

To identify direct targets of oncogenic TFs, we used chromatin immunoprecipitation by sequencing (ChIP-seq) to detect binding of estrogen receptor in breast cancer (*15*), androgen receptor and ETS Transcription Factor ERG (ERG) in prostate cancer (*16*), Transcription Factor 4 (Tcf4) and β-catenin in colorectal cancer (*17*), GLI Family Zinc Finger 1 (GLI1) and Paired Box 3 (PAX3) in rhabdomyosarcoma (*18*–*20*), and ETS transcription factor Fli-1 Proto-Oncogene (FLI1) in Ewing’s sarcoma (*21*) (fig. S1A). The number of peaks identified for each factor is indicated in fig. S1A. We also performed expression profiling analyses to identify genes whose expression is affected by the TFs (fig. S1B). The target genes were ranked on the basis of the number of different ChIP-seq peaks, peak heights, and their proximity to the transcription start site (TSS; measured as ranked distance; see Materials and Methods for details). Because of gene duplication events, humans often have many genes whose proteins have very similar if not identical functions. These proteins often are regulated differentially and could thus be targeted by different oncogenic TFs in different tumor types. To address this, we merged paralogous target genes to groups that are likely to have similar activities (fig. S2A and see Materials and Methods for details).

Significance of results was assessed by permutation analysis. Initial analysis revealed that the paralog group corresponding to Rho Associated Coiled-Coil Containing Protein Kinases and Large Tumor Suppressor Kinases (ROCK/LATS) was ranked first and the paralog corresponding to the *MYC* oncogenes—known targets of many oncogenic signals—was ranked third from all of the paralog groups (Fig. 1C). We also validated the result by restricting the set of genes analyzed to known regulators of the cell cycle in human cells (*25*) or human orthologs of *Drosophila* cell cycle regulators (*26*), which resulted in identification of *MYC*, *ROCK/LATS* kinase, and the cyclin-dependent kinase *CDK4/6/CCRK* paralog groups as common targets of lineage-specific oncogenic TFs (Fig. 1C). Analysis of the ChIP-seq peaks of the individual oncogenic TFs in the different tumor types showed that the signal was not derived from a single tumor type or an individual TF but represented broad-based regulation of the master regulatory genes by the oncogenic TFs (fig. S2B).

Similar analysis using genome-wide association data for risk of any type of cancer also identified five loci, including *MYC* genes and components of the CDK4/6 system (CDK inhibitors *CDKN2A/B*) (Fig. 1D). In addition, genome-wide association study (GWAS) identified human leukocyte antigen (HLA) and Telomerase Reverse Transcriptase (*TERT*) loci, which were not detected in the ChIP-seq–based analysis. This could be because they can affect tumorigenesis indirectly: TERT by causing chromosomal instability (*27*) and HLA by predisposing individuals for infection by tumor-causing viruses, such as human papillomavirus and Epstein-Barr virus (*28*, *29*). Together, two sets of completely orthogonal data, one derived from cell lines and another from patients, show that up-regulation of the CDK4/6 system and the MYC family of oncogenes are the predominant common cell-autonomous outcomes of the activation of cancer type–specific oncogenic TFs across many forms of human cancer.

### MYC also mediates the major shared transcriptional output of phosphorylation signaling

To identify common transcriptional mechanisms activated by phosphorylation signaling (Fig. 1E), we developed a set of cell lines that were either sensitive or resistant to common cytostatic kinase inhibitors. Cell lines were selected to represent major cancer types that have frequent kinase-activating mutations with known resistance mechanisms to treatments targeting the activated kinases. The 10 different parental cell lines used represented lung, colorectal, and breast carcinomas and chronic myeloid leukemia (CML) and were driven by hyperactivation of phosphorylation signals by mutations in B-Raf Proto-Oncogene (BRAF), RAS, epidermal growth factor receptor (EGFR), Erb-B2 Receptor Tyrosine Kinase 2 (ERBB2), Phosphatidylinositol-4,5-Bisphosphate 3-Kinase Catalytic Subunit Alpha (PIK3CA), and B cell receptor-Abelson Tyrosine-Protein Kinase 1 (BCR-ABL; table S2). Selected resistance mutations were introduced by CRISPR, and the cells were cultured with a drug concentration titrated to induce cell cycle arrest in the parental cell line. The proliferation of each parental cell line was robustly inhibited by the respective cytostatic drugs, consistent with regulation of the CDK system by the driver oncogenes. Single-cell clones were established from the cells that grew in the presence of the drug. In developing a resistant derivative of each cell line, we made use of the redundancy of the phosphorylation signaling, activating known alternate pathways that commonly rescue growth in the presence of a drug targeting a particular kinase (*30*). The drug treatment forces the resistant cell line to rely on the alternate pathway, thus enabling the investigation of common targets of different growth regulatory pathways.

We first treated a set of sensitive and resistant cell lines representing lung and colorectal cancer and CML with cytostatic kinase inhibitors targeting EGFR, mitogen-activated protein kinase (MAPK) kinase, and BCR-ABL. Cells treated with different drug concentrations for different times were labeled with DNA tags (Fig. 1F and see Materials and Methods), pooled, and subjected to single-cell RNA sequencing (scRNA-seq). Gene expression changes were modeled as a function of drug concentration, treatment duration, presence of resistance mutations, and cell cycle phase (see Materials and Methods). All drug treatments of the sensitive cell lines induced a clear cell cycle arrest–related transcriptional response (fig. S3A). To detect other responses, we determined the cell cycle phase for each cell and included it in the regression model to separate the direct effects of the drugs from indirect effects caused by cell cycle arrest, including G_1_ CDK signaling and transcriptional regulation by their downstream targets, the E2F family TFs. The genes regulated predominantly in the parental drug–sensitive cells by the different cytostatic drugs were highly overlapping between the different cell line pairs and heavily enriched in MYC targets (Fig. 1F and see Materials and Methods). Drug treatment resulted in G_1_ cell cycle arrest starting at 12 to 24 hours. In the resistant cell lines, cell cycle arrest was incomplete and/or required a higher drug dose (fig. S3A).

Comparison across the cell line pairs resulted in identification of genes that were regulated before the cell cycle arrest in a highly convergent manner in the different cell lines treated with distinct cytostatic drugs (table S2). The genes were heavily enriched in regulators of translation and ribosome biogenesis (Fig. 1G), consistent with previous observations that MYC regulates translation (*31*, *32*). The regulation of MYC preceded the down-regulation of its targets, suggesting that most shared transcriptional responses observed after the drug treatments were dependent on MYC (Fig. 1H and fig. S3B). Ribosome biogenesis factors, including nucleolar and coiled-body phosphoprotein 1 (NOLC1), were regulated with slightly faster kinetics on average than the rest of the MYC targets (Fig. 1H and fig. S3, B and D, 6-hour time point). Only a few of the genes appeared to be regulated independent of MYC. These included DUSP6, ETV4, and ETV5, which were down-regulated, and the negative regulator of ribosome biogenesis PNRC1 and two noncoding RNAs (MALAT1 and NEAT1), which were up-regulated. The early down-regulation of DUSP6 and MYC is most likely due to the corresponding mRNAs having a shorter half-life than the mRNAs of the other down-regulated genes (*33*) (fig. S3C).

The role of MYC in oncogenic signaling is well established (*34*, *35*), but what is unexpected is that MYC is almost exclusively driving the common, cell cycle–independent transcriptional response to kinase inhibitors. In summary, the results obtained with three complementary approaches indicate that most transcriptional consequences of oncogenic signaling that are common to multiple cancer types are either mediated by the E2F family of cell cycle regulators or by the MYC family of oncogenes.

### Common phosphorylation targets across human cancer types

**Fig. 2.**
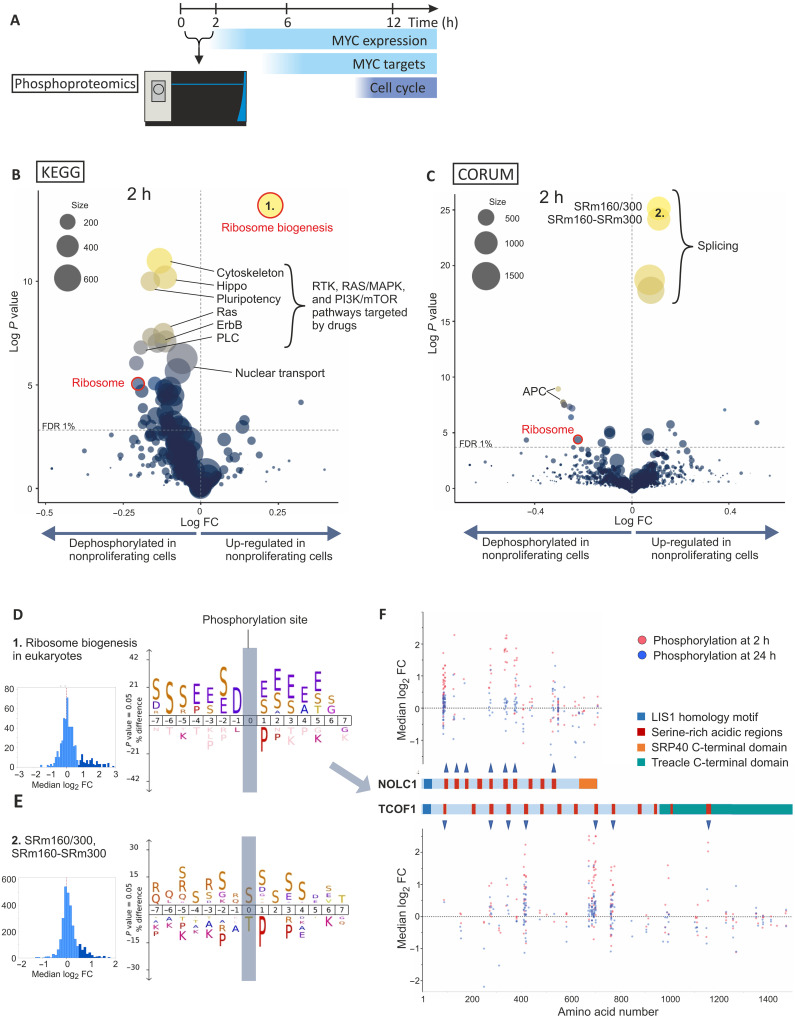
Phosphoproteomics profiling of shared targets of growth regulatory pathways. (**A**) Phosphoproteomics was performed at 30-min and 2-hour time points before widespread transcriptional changes or cell cycle arrest. (**B**) Average differential phosphorylation between proliferating and nonproliferating cells in all Kyoto Encyclopedia of Genes and Genomes (KEGG) pathways excluding the KEGG DISEASE category is shown as log fold change on *x* axis. Size of the circles indicates the number of phosphopeptides identified in the KEGG pathway. PLC, phospholipase C. (**C**) Average differential phosphorylation between proliferating and nonproliferating cells in all CORUM database protein complexes is shown as log fold change on *x* axis. Size of the circles indicates the number of phosphopeptides identified in protein complexes. (**D**) iceLogo motif analysis of the up-regulated phosphorylations (marked with darker blue in the histogram) compared to all phosphorylations (lighter blue) in the KEGG pathway ribosome biogenesis. (**E**) iceLogo motif analysis of the up-regulated phosphorylations (marked with darker blue in the histogram) compared to all phosphorylations (lighter blue) in the CORUM database entries SRm160/300 and SRm160-SRm300. (**F**) NOLC1 and TCOF1 phosphorylation changes (median log fold change) between nonproliferating (parental drug–sensitive cells) and proliferating cells (nontreated parental and resistant cells). *x* axis shows the position of the phosphorylation site in NOLC1 and TCOF1 proteins. Phosphorylated serine-rich acidic regions are indicated with an arrowhead. LisH, LIS1 homology. In (B) to (H), the data were generated from NCI-H1975, RKO, HCT116, LoVo (both resistance mechanisms), MCF-7 (temsirolimus, both resistance mechanisms), and KBM7/HAP1 (both drugs) cell lines as presented in table S2. In (B) and (C), *P* values were calculated using two-tailed one-sample *t* test, and dashed line indicates 1% Benjamini-Hochberg FDR.

In addition to transcriptional output, it is well established that posttranslational regulation has also more direct effects on protein activities [see, for example, (*36*–*38*)]. To study these nontranscriptional effects of growth signaling, we assessed the kinetics of the transcriptional changes and cell cycle arrest in our scRNA-seq data and performed proteomics analyses with ≤2-hour time points, before most of the transcriptional effects took place (Fig. 2A and fig. S3, A and B). On the basis of the scRNA-seq, cell cycle, and cell viability analyses, we selected drug concentrations that only caused cell cycle arrest in the drug-resistant derivative of each cell line. We performed phosphoproteomic characterization of the cell lines listed in table S2 using multistage mass spectrometry (MS^3^) with synchronous precursor selection (SPS) and tandem mass tags (TMTs), identifying more than 48,000 phosphopeptides in total (median, 19,771 per cell line) at <1% false discovery rate (FDR).

Analysis of common peptides, whose phosphorylation was altered by drug only in the parental cell lines but not in the corresponding resistant derivative, revealed multiple phosphorylation sites in proteins involved in metabolism, ribosome biogenesis, and translation. Several known phosphoregulatory sites were identified, validating our approach (fig. S4, A and B). In addition, we found many shared downstream phosphorylation events, many of which affected essential genes or genes linked to cell proliferation (table S3).

### Phosphorylation events target ribosome biogenesis before down-regulation of gene expression

The semirandom peptide detection inherent to MS using data-dependent acquisition results in missing data between sample subsets, which potentially limits the sensitivity of the analysis conducted on individual phosphopeptides. To improve the sensitivity, we next identified increases and decreases in phosphorylation affecting entire protein complexes or pathways (see Materials and Methods). Although we cannot rule out that other phosphorylation events are also associated with cell proliferation, this approach enabled the identification of several processes associated with proliferation across different cancer types. After drug treatment, we observed increased phosphorylation in the large nucleolar proteins involved in ribosomal RNA (rRNA) processing and splicing (Fig. 2, B and C, and fig. S4, C and D). This up-regulation occurred mostly in the acidophilic phosphorylation sites and serine clusters, suggesting a common mechanism for phosphorylation of these proteins (Fig. 2, D and E). In the case of ribosome biogenesis, these phosphorylations occurred mainly in the serine-rich acidic patches of the known rRNA synthesis factors Treacle protein (TCOF1) and its paralog NOLC1 (Fig. 2F). Notably, many of these sites have previously been shown to undergo pyrophosphorylation (fig. S5A) (*39*).

NOLC1 was also identified as one of the most prominent common MYC targets regulated transcriptionally by cytostatic drugs in our single-cell transcriptomics data (Fig. 1H and fig. S3, B and D), raising the possibility that it acts as a node integrating transcriptional and posttranslational signals. Therefore, we analyzed the regulation of NOLC1 and TCOF1 more closely (fig. S3D) and noticed that NOLC1 and TCOF1 also exhibited similar transcriptional responses to the drug treatments. Phosphorylation changes of both proteins occurred earlier than their transcriptional regulation. Increased phosphorylation in the acidophilic sites was short lived, whereas the dephosphorylation of several sites in the conserved C termini of these proteins persisted in the 24-hour time point (Fig. 2F and fig. S5, B and C). Ribosomal protein phosphorylation was reduced on average, and this change was also prominent at a later time point of 24 hours, together with down-regulated phosphorylation of ribosome biogenesis factors (fig. S5, B and C). Together, these findings suggest that the regulation of ribosomes is a key outcome of growth signaling in the studied cancer types.

### Protein interaction and metabolomic analysis identify common targets in ribosome biogenesis and metabolism

**Fig. 3.**
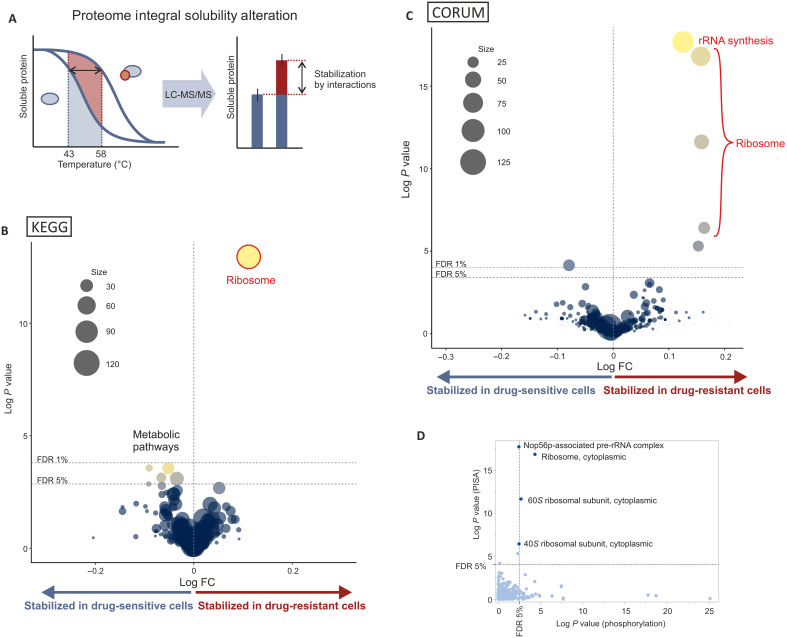
Protein interaction changes in proliferating cells. (**A**) PISA assay principle. (**B**) Volcano plot: Average differential thermal stability change in PISA assay between drug-resistant and -sensitive cells in all KEGG pathways excluding the KEGG DISEASE category is shown as log fold change on *x* axis. (**C**) Volcano plot: Average differential thermal stability change in PISA assay between drug-resistant and -sensitive cells in all CORUM database protein complexes is shown as log fold change on *x* axis. (**D**) One-sample *t* test *P* values for one-sample *t* tests of differential phosphorylation (*x* axis) and thermal stability (*y* axis) *P* values were calculated using a two-tailed one-sample *t* test. Dashed lines indicate 1 and 5% Benjamini-Hochberg FDR. Data were generated from NCI-H1975, RKO, and HCT116 cell line pairs, as presented in table S2, in five replicates for each condition. For each cell line pair, thermal stability change was calculated using the median of these replicates. The *t* tests were then calculated for the means of the thermal stability changes across all cell line pairs.

We next performed a proteome integral solubility alteration (PISA) assay (Fig. 3A) (*40*) in the same three adherent cell line pairs used in the scRNA-seq experiments to identify proteins, whose interactions change in response to cytostatic drugs. Consistent with the phosphorylation analysis, we detected a change in the soluble amounts of ribosome biogenesis regulators after 2 hours of drug treatment (Fig. 3, B and C, and fig. S6, A and B). Concurrent changes in phosphorylation and protein solubility in the same ribosome biogenesis–associated protein complexes (Fig. 3D) suggest that the observed phosphorylation events may regulate protein-protein interactions and ribosome stability. We also detected effects on metabolic pathways (Fig. 3B and fig. S6A).

**Fig. 4.**
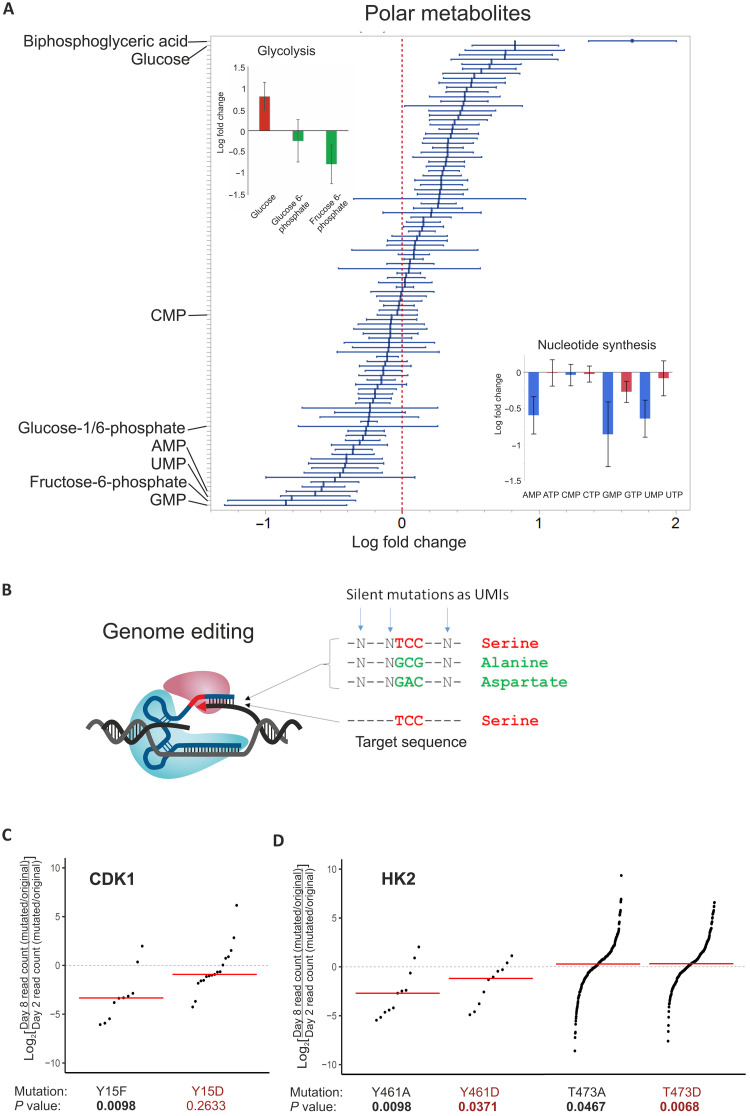
Metabolic changes in proliferating cells. (**A**) Quantification of polar metabolites by LC-MS/MS. Means ± SEM for eight cell line pairs is shown. Top left: Early glycolytic pathway metabolites glucose, fructose-6-phosphate, and fructose-1,6-bisphosphate. Bottom right: Nucleoside mono- and triphosphates. The data were generated from A549, NCI-H1975, RKO, HCT116, K562, MCF-7 (palbociclib), T47D, and KBM7/HAP1 (both drugs) cell lines as presented in table S2 and corresponding to phosphoproteomic samples. CMP, cytidine 5′-monophosphate; CTP, cytidine 5′-triphosphate; UTP, uridine 5′-triphosphate; GTP, guanosine 5′-triphosphate; ATP, adenosine 5′-triphosphate. (**B**) CGE assay. Phosphorylation sites were edited using prime editor or Cas9 with single-stranded oligodeoxynucleotides (ssODNs) as repair template for homology-directed repair. For each editing reaction, pegRNA or ssODN library consisted of sequences that restore the phosphorylatable residue, introduce nonphosphorylatable residue (alanine or phenylalanine), and introduce phosphomimetic residue (aspartate or glutamate). Additional silent mutations were introduced to adjacent codons to enable lineage tracing. UMI, unique molecular identifier. (**C**) Consistent with a previous publication (*43*), the effect of mutating Y15 phosphorylation site in the CDK1 gene on fitness of HAP1 cells. (**D**) The effect of mutating Y461 and T473 phosphorylation sites in the HK2 gene on the fitness of HAP1 cells. In (C) and (D), log_2_ ratios of day 8/day 2 are shown for each sequence tag pair after calculating the ratio of read counts for the mutated versus the original sequence at both time points. Red lines represent the median values, and *P* values from Wilcoxon signed-rank test are shown for each experiment.

To further characterize the metabolic state of the cells after drug treatment, we performed metabolomics analysis using liquid chromatography (LC)–MS. A targeted characterization of polar metabolites in selected cell lines 24 hours after drug treatment revealed a decrease in the nucleotide monophosphates—uridine 5′-monophosphate (UMP), adenosine 5′-monophosphate (AMP), and guanosine 5′-monophosphate (GMP)—and altered glycolytic flux based on accumulation of glucose and bisphosphoglycerate and decrease in fructose-6-phosphate (Fig. 4A) that mainly occurred in the drug-sensitive cells. We also observed inhibition of de novo nucleotide synthesis based on a decrease in levels of UMP, the key intermediate for synthesis of pyrimidine nucleotides via the de novo pathway (Fig. 4A, bottom right).

The accumulation of glucose suggested that the impairment in glycolysis could be due to decrease in activity of hexokinase, an enzyme that controls one of the rate-limiting steps of glycolysis. The expression of the gene for hexokinase 2 (HK2) was also moderately down-regulated by the chemotherapeutic drugs (fig. S7). However, the relatively rapid changes in glycolytic metabolites, and the solubility change of components of metabolic pathways already at 2 hours (Fig. 3B) suggested that some effects on metabolism could be mediated by direct posttranslational regulation. We therefore tested whether phosphorylation of HK2 contributed to the observed effects. We measured the requirement of known HK2 phosphorylation sites using competitive precision genome editing (CGE) assay (Fig. 4, B to D) (*41*). CGE assay uses silent mutations as sequence tags that enable lineage tracing (Fig. 4B). Although variance between lineages is high because of a large number of measured lineages, the aggregate statistical power of CGE is very high because the outlier lineages have a smaller effect on the median than the sum of the lineage read counts (*41*, *42*). Therefore, it can be used to measure small differences with robust confidence estimates using a relatively short dropout experiment, where the effect size is still limited. We did not detect an effect on cell proliferation by mutation of HK2 residue T473, whose phosphorylation has been reported to affect HK2 activity (*43*, *44*). However, this phosphorylation is not found in the publicly available MS datasets, whereas phosphorylation in the nearby tyrosine residue (Y461) is commonly observed (*45*). Mutation of Y461 in HK2 inhibited growth of HAP1 cells (Fig. 4D), implicating phosphorylation of Y461 in regulation of HK2 activity. These results establish that transcriptional control and posttranslational regulation act in concert to regulate both ribosome biogenesis and metabolism.

### NOLC1 and TCOF1 define the proliferative compartment in human cancer

**Fig. 5.**
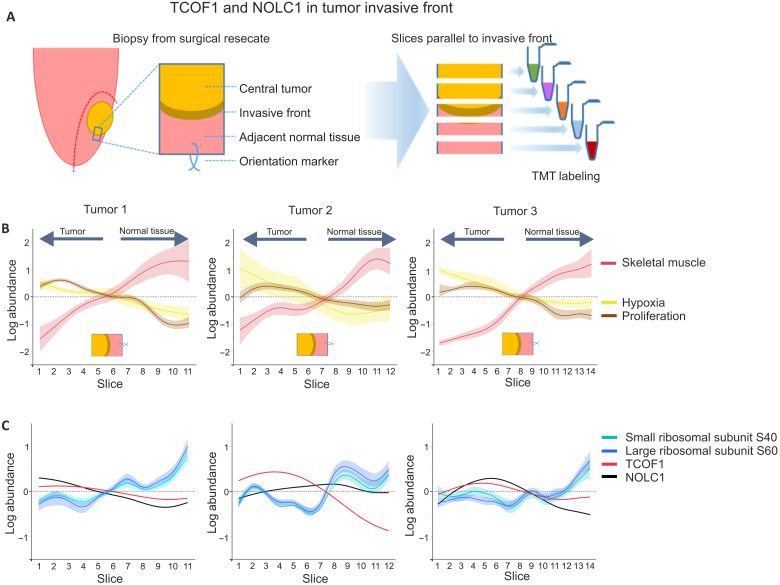
Proliferation associated changes in HNSCC invasive front. (**A**) HNSCC invasive front biopsies. Biopsies were collected from lateral tumors of the tongue following hemiglossectomy. Each biopsy was cut into 500-μm slices parallel to the invasive front, and slices of the same tumor were analyzed as one TMT multiplex. (**B**) Expression of hypoxia markers [Solute Carrier Family 2 Member 1 (GLUT1) and Pyruvate Dehydrogenase Kinase 1 (PDK1)], skeletal muscle–specific proteins (*49*), and proliferation markers (KI-67, PCNA, and MCM2-7) in the invasive front biopsies. Smoothing spline fit with 95% confidence interval shaded is shown. Tumor-normal tissue interface is marked by a transition from proliferation and hypoxia marker expression to skeletal muscle–specific protein expression. Proliferation marker expression is highest in the invasive front and decreases toward central tumor, whereas hypoxia marker expression is highest in the more central tumor. (**C**) Expression of small and large subunit ribosomal proteins and ribosome biogenesis factors NOLC1 and TCOF1 in the invasive front. Smoothing spline fit is shown. For ribosomal proteins, 95% confidence interval for the fit is shaded.

Our results described above establish that in addition to the cell cycle, the main processes that associate with cell proliferation in different types of cultured human cancer cells are ribosome biogenesis and translation. To determine whether this correlation is also observed in human cancer, we performed proteomic analyses of squamous cell carcinoma (SCC) of the tongue, a model that allows clear separation of proliferative and nonproliferative compartments of genetically similar cancer cells (Fig. 5A). In oral SCCs, cells in the inferior border of the tumor, the invasive front, have high proliferative activity (*46*, *47*). Comparison of the invasive front and the central tumor enabled the identification of processes that are specific to proliferating cancer cells.

Biopsies were collected from patients with tongue cancer treated with hemiglossectomy, which results in a large surgical margin enabling the collection of a biopsy spanning the invasive front (Fig. 5A). Fresh frozen biopsies were cut into 500-μm sections parallel to the invasive fronts, enriching the invasive front cells to specific sections. Protein expression specific to proliferating cells was identified by correlation profiling (*48*), where distributions of proteins across the slices were compared to known proliferation markers [Marker Of Proliferation Ki-67 (KI-67), Minichromosome Maintenance Complex Components 2-7 (MCM2-7), and proliferating cell nuclear antigen (PCNA)] (*46*, *47*). The proliferation markers were highly enriched in the sections containing the invasive front (Fig. 5B). On the other hand, adjacent normal tissue exhibited high expression of proteins specific to skeletal muscle cells (*49*), as would be expected from the cells of the tongue (Fig. 5B). TCOF1 and, to a lesser extent, its paralog NOLC1 exhibited an expression profile similar to proliferation markers, with the highest expression in the tumor invasive front, whereas ribosomal protein expression was highest in the adjacent normal tissue (Fig. 5C). This suggests that while both proliferating tumor cells and muscle cells require high protein synthesis capacity, the regulation of ribosome production has distinct, potentially therapeutically exploitable, characteristics in proliferating cancer cells, exemplified by elevated expression of TCOF1 and NOLC1.

### Phosphorylation of NOLC1 is required for cancer cell proliferation

Our results establish that the ribosome biogenesis regulators including NOLC1 and TCOF1 correlate with cell proliferation both in cultured cells representing multiple cancer types and in human oral SCC tumors. The coordinated changes in phosphorylation and protein solubility that correlate with cell proliferation suggest that at least some of the events might be mechanistically important for driving cell proliferation in response to phosphorylation signaling. To test this, we selected a set of phosphorylation sites, including NOLC1 T607 and T610 and TCOF1 S1410, for detailed functional validation using CGE assay based on the following criteria: (i) essentiality of the protein, (ii) conservation of the phosphorylation site, (iii) robust detection of the phosphopeptide in multiple different cancer types, and (iv) consistent regulation between proliferating and nonproliferating cells (fig. S8). On the basis of the recent analysis by Johnson *et al.* (*50*), the selected sites are phosphorylated efficiently by multiple kinases, which is consistent with the notion that they are targets of multiple growth regulatory pathways. We next measured the requirement of the phosphorylation sites using the CGE assay.

**Fig. 6.**
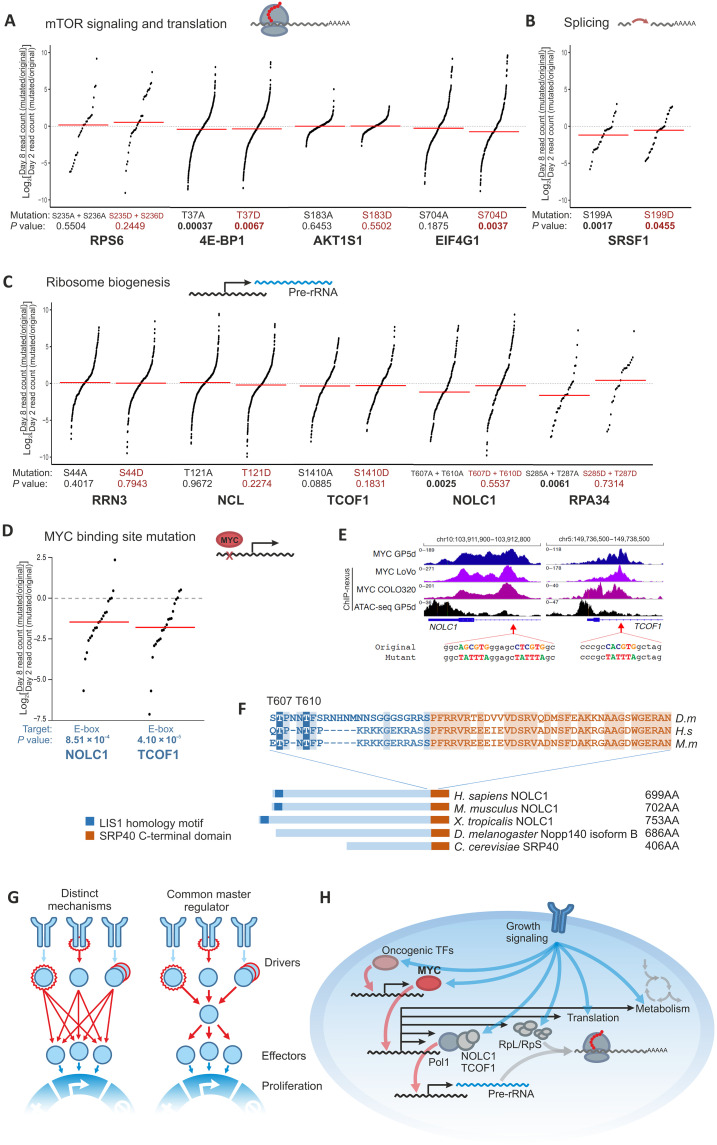
Fitness effect of selected phosphorylation site mutations. (**A**) CGE assay results for mutating phosphorylation sites in effectors of mTOR signaling and translational regulators. (**B**) CGE assay results for mutating splicing factor Serine And Arginine Rich Splicing Factor 1 (SRSF1) phosphorylation site S199 on the fitness of HAP1 cells. NCL, Nucleolin. (**C**) CGE assay results for mutating phosphorylation sites in ribosome biogenesis factors on the fitness of HAP1 cells. (**D**) CGE assay results for mutating E-boxes in the promoters of NOLC1 and TCOF1 on the fitness of HAP1 cells. (**E**) Promoter regions of NOLC1 and TCOF1. MYC ChIP-nexus traces in three colorectal cancer cell lines, GP5d, LoVo, and COLO320 are shown in purple and ATAC-seq in GP5d cells in black. Targeted E-boxes and mutations introduced by CGE are shown below the traces. (**F**) Interpro domain annotations of NOLC1 paralogs in *H. sapiens* (*H.s*), *Mus musculus* (*M.m*), *Xenopus tropicalis*, *Drosophila melanogaster* (*D.m*), and *Saccharomyces cerevisiae* and alignment of the sequence containing the phosphorylation sites T607/T610 and N-terminal half of the highly conserved SRP40 C-terminal domain in *H. sapiens*, *M. musculus*, and *D. melanogaster*. Conserved residues between fly and mammals are indicated with shaded background. Phosphorylations of the residues corresponding to human T607 and T610 have been observed in *D. melanogaster* and *Drosophila yakuba*, respectively (*52*). (**G**) Alternative models for regulation of cancer cell proliferation. It is more likely that different upstream driver mutations engage effectors needed for cell proliferation via a common master regulator (right) than via distinct molecular mechanisms (left). (**H**) Schematic presentation of common targets of transcriptional and posttranslational growth regulatory mechanisms. RpL, large ribosomal subunit proteins; RpS, small ribosomal subunit proteins; Pol1, polymerase I. In (D) to (F), log_2_ ratios of day 8/day 2 are shown for each sequence tag pair after calculating the ratio of read counts for mutated versus restored wild-type sequence at both time points. Red lines represent the median values, and *P* values from Wilcoxon signed-rank test are shown for each experiment.

Control experiments established that some phosphorylation events that correlate with cell proliferation are not causative. For example, mutation of well-studied phosphorylation sites (S235 and S236) of ribosomal protein S6 had no effect, consistent with earlier observations (*51*) (Fig. 6A). Analysis of phosphorylation sites in other proteins involved in translation and mechanistic target of rapamycin (mTOR) signaling also revealed that a mutation of S183 in AKT1 Substrate 1 (AKT1S1) had no effect. A minor effect was detected by mutation of another classical phosphorylation site, Eukaryotic Translation Initiation Factor 4E Binding Protein 1 (4E-BP1) T37 (Fig. 6A). We also found that a phosphomimetic mutation of S704 in Eukaryotic Translation Initiation Factor 4 Gamma 1 (EIF4G1) decreased cell proliferation (Fig. 6A), consistent with the up-regulation of S704 phosphorylation in response to cytostatic drugs (fig. S8).

Stronger effects were detected by mutating phosphorylation sites in proteins involved in two other processes, splicing (Fig. 6B) and ribosome biogenesis (Fig. 6C), that were rapidly modulated by cytostatic drugs. In particular, phosphorylation sites in two regulatory components of RNA polymerase I, NOLC1 and RNA Polymerase I Subunit G (RPA34), had a strong negative impact on cell proliferation (Fig. 6C). Notably, both NOLC1 and its paralog TCOF1 were also regulated by MYC, and mutation of the MYC binding site in the promoter of either gene also caused a decrease in cell proliferation (Fig. 6, D and E), establishing that NOLC1 regulation at both the transcriptional and posttranslational level is required for cell proliferation. Notably, the phosphorylation of T607 in NOLC1 is conserved all the way to invertebrates such as *Drosophila* (Fig. 6F) (*52*), suggesting that the identified mechanism of regulation is conserved across species.

## DISCUSSION

Intuitively, the diversity of driver oncogenes and the heterogeneity of individual tumor cells suggest that the downstream regulatory network of cancer would have a very highly complex structure, which would vary extensively between cancer types and between cell clones or even individual cells within a tumor. However, as a large number of genes need to be precisely controlled to drive cell proliferation and division, it is unlikely that all upstream drivers would use distinct molecular mechanisms to target the same set of effector genes (Fig. 6G). Here, we hypothesized that driver genes would regulate a smaller set of master regulators of the cancer phenotypes. To identify these master regulators in an unbiased and comprehensive manner, we investigated the common outcomes of oncogenic transcriptional and posttranslational regulation in major forms of human cancer that constitute 46% of the worldwide cancer mortality (*53*). We found that diverse types of upstream oncogenes, including the protein kinases and TFs that contribute to major forms of human cancer converge to activate a single common downstream growth regulatory process. Our findings indicate that the gene regulatory network shared by many cancers has an hourglass shape, with a large number of potential driver genes converging on a small number of master regulators and then diverging again into a large number of effector genes that underpin the cancer phenotype. Unlike the upstream drivers, which are typically tumor type specific, the master regulators and their effector genes appear to be common to many major forms of cancer. Because of the diversity of cancer types, we cannot rule out the possibility that some tumor types use downstream mechanisms different from those identified here. However, of the remaining tumor types that contribute a substantial fraction to overall cancer mortality, pancreatic cancer is commonly driven by RAS/MAPK pathway, and ovarian cancers harbor frequent MYC amplifications. Therefore, we expect that our findings can be generalized to the majority of human cancers.

The concept of master regulator has been proposed previously by others (*54*–*56*). For example, Paull *et al.* (*57*) used computational approaches to identifying more than 400 master regulators from The Cancer Genome Atlas transcriptomics data encompassing nearly 10,000 samples from multiple cancer types. The large number of master regulators identified by this approach reflects the fact that it is very difficult to determine the sequence of regulatory events from static transcriptomics data. Although Paull *et al.* (*57*) identified MYC and E2F1 as some of the most prominent among many master regulators, they also classified many proteins acting either upstream or downstream of these key regulators as master regulators. In contrast, our approach considers direct TF binding (e.g., ChIP-seq) and uses time-series data (Fig. 1H and fig. S3B), enabling inference of direct regulatory events and decoupling growth regulation from cell cycle phase–specific gene expression (single-cell transcriptomics time course). As a consequence, our approach results in identification of far fewer key master regulators than methods based on static transcriptomes.

Of the commonly recognized 10 cancer hallmarks (*23*), our approach detected five: (i) growth/proliferation and (ii) evasion of growth suppression (MYC and CDK4/6/E2F axis), (iii) immortality (TERT), (iv) DNA damage, and (v) immune mechanisms. The immune signal was an association between HLA locus variants and cancer; this association is mainly derived from a small subset of cancers that share an infectious etiology. The absence of common regulatory mechanisms for other cancer hallmarks could be due to posttranslational regulation distinct from phosphorylation or tissue-specific mechanisms. Consistent with the tissue specificity model, it is well established that different tumor types preferentially metastasize to different organs, and only some of this can be explained by anatomical considerations (*58*). Regarding invasion, some recent evidence from intestinal tumors suggests that some aspects of invasion could also be driven by tissue-specific mechanisms. In particular, Adenomatosis Polyposis Coli Tumor Suppressor (APC) null intestinal epithelial cells secrete the WNT inhibitor NOTUM, which induces differentiation of normal stem cells (*59*). Growth of APC null tumors secreting NOTUM induces a transcriptional response similar to that induced by tissue damage in neighboring normal tissue (*60*), suggesting that invasion of tumors may depend on damaging normal tissue and aberrant wound repair mechanisms. Consistent with tissue specificity of invasion, metastases can also show less invasive phenotype than primary tumor. For example, invasiveness of liver metastases of colorectal cancer and melanoma are not fully defined by the invasiveness of the primary tumor; the metastases can be surrounded by microcapsule-like structure or a complete ring of fibrosis isolating them from the liver, reminiscent of structures that commonly form around benign tumors (*61*, *62*). This hypothesis that cancer hallmarks could be divided to those having common mechanisms and those acting in a tissue-specific manner warrants further study.

The main growth regulatory processes activated by the oncogenic mechanisms analyzed here are metabolism, translation, and ribosome biogenesis (Fig. 6H). Translation is affected both through phosphorylation of multiple components of the translation machinery and by transcriptional control of multiple initiation factors by the master regulator MYC. Similarly, in ribosome biogenesis, the direct effects of phosphorylation signaling appear to affect proteins such as RPA34 and NOLC1 that are involved in ribosomal RNA synthesis, whereas the indirect action via MYC leads to up-regulation of multiple proteins involved in ribosome biogenesis. MYC is known to increase protein synthesis by inducing expression of multiple genes involved in ribosome biogenesis and translation (*31*, *32*, *63*, *64*). Our findings show that the transcriptional control by MYC and posttranslational regulation by oncogenic kinase signaling act in concert to increase both the specific activity and concentration of components of protein synthetic machinery. In particular, we identified a key downstream node, NOLC1, whose activity is regulated both transcriptionally and posttranslationally. Reverse genetic experiments using CGE established that both types of regulation of NOLC1 are independently required for cell proliferation, highlighting the importance of this process (Fig. 6H).

The identified feed-forward loop targeting NOLC1 has implications to the known phenomena of oncogene cooperation. It is well established that MYC cooperates with RAS in cellular transformation. Our results suggest that the cooperative effect is quantitative in nature, where neither RAS/MAPK nor MYC alone can optimally activate NOLC1, as both transcriptional and posttranslational regulation is required. Given that different tissues and cell types have different endogenous levels of RAS/MAPK and MYC activity (*45*), our results also suggest that in a given cell type, either MYC or RAS/MAPK would be rate limiting for tumorigenesis. This quantitative effect could thus also contribute to the phenomenon called cancer hyperbola, i.e., the tendency of different cancer types to have either frequent copy number alterations (that could activate MYC) or point mutations (that commonly activate kinases) (*65*, *66*).

The primary role of ribosome biogenesis for human cell proliferation and cancer is similar to simpler organisms, such as *Escherichia coli* and yeast, where cell growth rate and ribosome concentration are linearly correlated (*67*, *68*). Growth signals lead to an initial increase in ribosome biogenesis, followed by broader protein synthetic and anabolic activity. Our results suggest that multicellular organisms have evolved additional control mechanisms to the old circuit that drives growth in response to nutrients. These mechanisms limit growth by hierarchical organization of cells into stem cells and differentiated cells (*69*) and by cell-to-cell signaling mechanisms that are required to specify or reinforce the proliferative state of specific cell types within particular tissues.

Identification of tumor type–specific oncogenic drivers has enabled development of “mechanism-based” antineoplastic agents that target specific upstream processes activated in particular tumor types, for example, by directly binding to a single oncogenic protein kinase (*70*). These targeted drugs are safe and effective but, in almost all cases, lead to development of resistance due to cancer heterogeneity—the presence of resistant subpopulations harboring mutations that prevent drug binding or activating another upstream pathway driving the regrowth of the tumor. Despite the extreme heterogeneity of individual cancer cells that facilitates resistance to therapy via rewired upstream pathways or mutated driver genes, our findings show that behind all the complexity, there is a molecular commonality of downstream mechanisms shared by many forms of human cancer. Elevated MYC expression has been associated with resistance to various therapies in many cancers, including some that were not covered by our study, suggesting that our findings on the convergence of oncogenic transcription to MYC can be generalized to other forms of human cancer (*34*).

Our findings have important implications for cancer therapy and prevention, as they suggest that it is possible to develop a novel class of broad-spectrum antineoplastic agents that do not have the severe limitations of current chemotherapies. Existing antimetabolites and conventional chemotherapeutics are severely toxic, in part because they do not target specific proteins or pathways activated in tumors. Instead, they nonspecifically damage DNA and/or target multiple processes that are related to but not identical to the mechanisms uncovered here. For example, the commonly used chemotherapeutic 5-fluorouracil not only targets thymidylate synthase, an enzyme that acts in de novo thymidylate synthesis pathway, but also causes DNA damage due to misincorporation of 5-fluoro-2′-deoxyuridine into DNA (*71*). Our results suggest that compounds that decrease the level of ribosome biogenesis or increase ribophagy, such as RNA polymerase I inhibitors (*72*, *73*), would more directly target a key consequence of oncogenic mutations. Furthermore, given the fact that genetic variants that decrease the activity of the pathways identified here decrease cancer risk but have no known harmful effects (Fig. 1D), a low-dose or partial antagonist targeting the pathways might act as a chemopreventive agents, decreasing the incidence of a broad spectrum of human cancers.

## MATERIALS AND METHODS

### Cell lines

HAP1 (#C631, RRID:CVCL_Y019) and KMB-7 (#C628, RRID:CVCL_A426) cell lines were obtained from Horizon Discovery and maintained in low-density cultures in Iscove’s modified Dulbecco’s medium according to the vendor’s guidelines. RKO (RRID:CVCL_0504), HCT116 (RRID:CVCL_0291), LoVo (RRID:CVCL_0399), BT-474 (RRID:CVCL_0179), T47D (RRID:CVCL_0553), MCF-7 (RRID:CVCL_0031), NCI-H1975 (RRID:CVCL_1511), A549 (RRID:CVCL_0023), K562 (RRID:CVCL_0004), CRL-2061 (RRID:CVCL_0041), VCaP (RRID:CVCL_2235), and SK-N-MC (RRID:CVCL_0530) cells were purchased from American Type Culture Collection and cultured according to the vendor’s guidelines in the medium specified by the vendor. To sensitize MCF-7 cells to temsirolimus, cells were cultured in reduced fetal bovine serum (FBS; 5%) and without insulin. Before ChIP, MCF-7 cells were hormone starved for 48 hours and subsequently mock-treated (minus ligand) or stimulated for 1 hour with 100 nM estradiol (E2). GP5d cells were obtained from Sigma-Aldrich (95090715) and cultured in Dulbecco’s modified Eagle’s medium (DMEM) supplemented with 10% FBS, 2 nM l-glutamine, and 1% penicillin-streptomycin. Wild-type and Myc-null Rat1 fibroblasts (*74*) were a gift from J. Sedivy, Brown University, and R. Bernards, Netherlands Cancer Institute. The cells were maintained in DMEM with 10% FBS and antibiotics.

Phosphatase and Tensin Homolog (PTEN) knockout (RKO, HCT116, LoVo, BT-474, NCI-H1975, and A549), RB Transcriptional Corepressor 1 (RB1) knockout (T47D and MCF-7), Neurofibromin 1 (NF1) knockout (MCF-7 and K562), and PIK3CA H1047R mutant (LoVo) cell lines were generated using Alt-R CRISPR-Cas9 from Integrated DNA Technologies according to the vendor’s guidelines. Briefly, crRNA and tracrRNA duplex complexed with Cas9-HiFi protein was transfected using CRISPRMAX (Invitrogen). Transfection was verified after 2 days by imaging the fluorescence from the atto550 label in the tracrRNA. Single-stranded oligod-eoxynucleotide (ssODN) was used as a homology-directed repair template for PIK3CA H1047R mutation (table S4) at 3 nM concentration. All crRNA sequences are listed in table S4. Resistant cells were selected by culturing with a drug concentration titrated to induce cell cycle arrest in the parental cell line. Single-cell colonies were cultured from cells that grew in the presence of the drug, and resistance mutation was verified by Sanger sequencing. Unless stated otherwise, drug concentrations used in the experiments were as follows: RKO, 20 nM trametinib; HCT116, 8 nM trametinib; LoVo, 20 nM trametinib; BT-474, 20 nM lapatinib; NCI-H1975, 100 nM osimertinib; A549, 100 nM trametinib; T47D, 200 nM palbociclib; MCF-7, 200 nM palbociclib or 20 nM temsirolimus; KBM-7/HAP1, 300 nM imatinib or 80 nM trametinib; K562, 300 nM imatinib.

### Human participants

Collection of head and neck SCC (HNSCC) invasive front samples was approved by the Ethics Committees of Southwest Finland (ETMK 166/1801/2015) and Turku University Central Hospital (TO6/022/17) and was conducted according to the principles of Declaration of Helsinki. Informed consent was obtained from the participants. Biopsies were taken from the resected tumors without compromising the routine diagnostics, and the orientation of the biopsy was marked with a stitch before snap-freezing the sample. The participants’ sex, gender, race, ethnicity, or age is not reported because the analyses compare different regions of the same tumor, and there are no conclusions on the differences between patients.

### Chromatin immunoprecipitation by sequencing

Antibodies to Tcf4 (clone 6H5-3, Exalpha Biologicals), β-catenin (rabbit polyclonal antibody: H-102, Santa Cruz Biotechnology, RRID:AB_634603), PAX3 (rabbit polyclonal antibody, catalog no. CA1010, Calbiochem), GLI1 (rabbit polyclonal antibody, H-300, Santa Cruz Biotechnology, RRID:AB_2111764), estrogen receptor (rabbit polyclonal antibody, HC-20, Santa Cruz Biotechnology, RRID:AB_631471), p300 (rabbit polyclonal antibody, N-15, Santa Cruz Biotechnology, RRID:AB_2293429), RNA polymerase II (rabbit polyclonal antibody, H-224, Santa Cruz Biotechnology, RRID:AB_2268548), α-H3K4me1 (rabbit polyclonal antibody, ab8895, Abcam, RRID:AB_306847), and normal immunoglobulin G (IgG) (mouse, sc-2025; rabbit, sc-2027; Santa Cruz Biotechnology, RRID:AB_737182 and RRID:AB_737197) were used in ChIP. ChIP-seq was performed as described in Tuupanen *et al.* (*75*)

The ChIP-seq data were analyzed as described in Wei *et al.* (*76*). Sequencing reads were mapped to the NCBI36 release of the human genome using Maq version 0.6.5 (*77*). Only reads with a mapping quality score of ≥30 were accepted. Reads were then extended to estimated fragment length, and peak height was determined at each position as the number of overlapping extended reads. For each peak of height eight or more, the total number of sequences in the continuous region of four or more overlapping sequences was compared to the number of sequences in the same region in the IgG control. The probability of observing the difference between the sequence counts in the ChIP sample and IgG control by chance was estimated using the Winflat program (*78*), and peaks that had probability smaller than 0.05 (without multiple hypothesis testing correction) were selected for further analysis.

### Small interfering RNA treatment and expression profiling

For small interfering RNA (siRNA) knockdown, predesigned FlexiTube siRNAs (QIAGEN) for the TFs targeted in ChIP, as well as control siRNA (SI03650325, QIAGEN), were transfected into cells using HiPerFect Transfection Reagent (catalog no. 301704, QIAGEN). Transfections were performed in two steps with 24-hour interval, and the cells were harvested 48 hours after the transfections. Expression profiling of endoplasmic reticulum was done by adding either 100 nM estradiol (E2) or mock ligand (ethanol) to MCF-7 cells 4 hours before lysis, and expression profiling of Androgen Receptor (AR) by adding 1 nM synthetic androgen methyltrienolone to VCaP cells. The cells were hormone starved for 48 hours before ligand addition. Total RNA were prepared by QIAshedder (catalog no. 79656, QIAGEN) and QIAGEN RNeasy Kit (catalog no. 74104). Expression profiling was performed using Affymetrix human genome U133plus2.0 arrays. The background noise was then corrected according to the Robust Multi-Array expression measure using sequence information (GCRMA) method (*79*). The probe sets were subsequently filtered for expression measurements to have a value of 100 fluorescence units in at least 25% of the samples. Differential expression analysis was performed by fitting a linear model as described in the Limma package (*80*); *P* values were adjusted according to Benjamini and Hochberg’s method to control the FDR. Probes showing significant differences (*P* < 0.01) between control samples were eliminated from the analysis.

### GWAS variant information

All single-nucleotide polymorphisms (SNPs) that were associated either with the trait “cancer” or with a trait that had cancer, as a parent trait were downloaded from the GWAS catalog (date: 19 November 2021) (*81*). There were 7796 SNPs associated with at least one of the cancer traits and 11,155 cancer trait associated with one of the SNPs.

### Single-cell RNA sequencing

CD298 and β_2_-microglobulin antibodies from BioLegend (341711, RRID:AB_2876646 and 316302, RRID:AB_492835) were labeled with TotalSeqB-oligos according to a protocol by van Buggenum *et al.* (*82*). Cells were cultured on 24-well plates and treated with varying drug concentrations and durations. In the end of the drug treatment, each row and column on the plate was labeled according to the cell hashing protocol by Satija laboratory (https://cite-seq.com/protocols/), with a unique TotalSeqB barcode, resulting in unique combination of two barcodes for each treatment condition. After the labeling, cells from the whole plate were pooled and resuspended in phosphate-buffered saline (PBS), and sequencing libraries were prepared on a Chromium platform using Single Cell 3′ v3 and feature barcoding reagent kits (10x Genomics, Pleasanton, CA) according to the manufacturer’s instructions. For each plate, 2500 to 5000 cells were sequenced at a depth of 50,000 to 100,000 reads per cell on a NovaSeq (Illumina). Preprocessing of the data was performed using CellRanger v3.0.2 (10x Genomics). Cells were further filtered on the basis of read count and TotalSeqB barcode count distribution that did not match a single to a treatment condition to remove probable empty beads. Gene expression was modeled as a function of drug concentration, treatment duration, cell cycle phase, and presence of resistance mutation, and genes were ranked by the explanatory power of the drug effect in the model.

### Phosphoproteomics

Cell line samples were collected at early (30 min or 2 hours) and late (24 hours) treatment time points. The cells were washed twice with ice-cold PBS and scraped into cold PBS with phosphatase inhibitors (PhosSTOP, Roche). Pelleted cells were snap-frozen and lysed with 8 M urea buffer with 100 nM Triethylammonium bicarbonate (TEAB; Sigma-Aldrich). Lysates were reduced with dithiothreitol at a final concentration of 20 mM and alkylated with indole-3-acetic acid at a final concentration of 40 mM. For digestion, the buffer was diluted to <2 M urea concentration, and samples were digested overnight with Lys-C (Wako), followed by 4-hour digestion with Trypsin (Promega) at room temperature. Digests were desalted, and 200 to 400 μg of each sample was labeled with TMT or TMTPRO reagents. Multiplexed samples were reverse-phase fractionated at high pH on an Acquity ultraperformance LC (UPLC) system (Waters) similarly to Christoforou *et al.* (*83*). Ten percent of each fraction was taken for total protein analysis, and the remaining was enriched with a modified Sequential elution from Immobilized Metal Affinity Chromatography (SIMAC) procedure. Briefly, dried peptides were reconstituted with 50% acetonitrile (ACN) and 0.1% trifluoroacetic acid (TFA) and incubated with Titansphere beads (Hichrom Ltd.) loaded in equal volume in 80% ACN, 5% TFA, and 1 M glycolic acid for 30 min with shaking (≥4:1 bead:peptide ratio). Beads were washed once with 80% ACN and 1% TFA and once with 10% ACN and 0.1% TFA and eluted with ~1.2% ammonia after 10-min incubation with shaking. Supernatants from loading and wash steps were pooled for five to six fractions, and each pool was dried and enriched with High-Select Fe-NTA Phosphopeptide Enrichment Kit (Thermo Fisher Scientific).

The LC–electrospray ionization (ESI)–MS/MS analyses were performed on a nanoflow high-performance LC (HPLC) system (Easy-nLC1000, Thermo Fisher Scientific) coupled to the Orbitrap Fusion Lumos mass spectrometer (Thermo Fisher Scientific, Bremen, Germany) using SPS-MS^3^ acquisition method. Kyoto Encyclopedia of Genes and Genomes (KEGG) pathway data (January 2022 release) (*84*), excluding KEGG DISEASE category, were obtained using KEGGREST R package. Protein complexes were downloaded from CORUM database (3.9.2018 release) (*85*). Phosphorylation site motif analysis was performed using iceLogo (*86*).

Frozen tumor biopsies were cut into 500-μm slices, and each slice was pulverized with a dounce in a microcentrifuge tube. Pulverized samples were lysed, reduced, alkylated, and desalted similar to cell line samples. Each slice (100 μg) was labeled with TMTPRO, and pooled sample was offline fractionated with Agilent 1260 HPLC system following a protocol by Mertins *et al.* (*87*). Fractions were pooled to 12 samples, and 10% of each fraction was taken for total protein analysis. Remaining sample was enriched with High-Select Fe-NTA Phosphopeptide Enrichment Kit (Thermo Fisher Scientific). The LC-ESI-MS/MS analyses were performed on a nanoflow HPLC system (Easy-nLC1200, Thermo Fisher Scientific) coupled to the Orbitrap Fusion Lumos mass spectrometer (Thermo Fisher Scientific, Bremen, Germany) equipped with a nanoelectrospray ionization source and FAIMS interface (Thermo Fisher Scientific).

### Protein interaction analysis by PISA assay

PISA assay was performed as described previously (*40*). Briefly, samples were collected at 2- and 24-hour treatment time points. Each resistant/sensitive cell line pair was analyzed in five replicates for drug-treated and control cells split between two TMT 11-plexes with a calibrator sample. TMT multiplexes were cleaned and desalted using on C18 SepPack column, fractionated by reversed-phase chromatography at high pH using capillary flow rate of 200 μl/min and a binary solvent system consisting of 20 mM NH_4_OH in H_2_O and 20 mM NH_4_OH in ACN. The elution was monitored measuring ultraviolet absorbance at 214 nm. A total of 96 fractions, 100 μl each, were collected, concatenated into 24 fractions, dried, and analyzed on Thermo Orbitrap Q-Exactive HF coupled to UltiMate 3000 RSLC nanoUPLC system. The data were normalized by median, and the data between different TMT multiplexes were combined by calculating a ratio to the calibrator sample included in both multiplexes. Drug-induced changes were identified by comparing the treated cells to nontreated cells, and proliferation-associated changes were identified by comparing the drug-induced changes between sensitive and resistant cells. Protein solubility changes in KEGG pathways and protein complexes were analyzed similarly to the phosphoproteomics data.

### Metabolite analyses

Samples were collected after 24-hour drug treatment, and intracellular metabolites were extracted using a methanol/chloroform method. Briefly, 600 μl of methanol/chloroform (2:1, v/v) was added to each cell pellet (~50 μl). Samples were vortex mixed and sonicated for 15 min. Two hundred microliters of chloroform and water were added, and samples were vortex mixed and separated by centrifugation at 17,000*g* for 15 min. The aqueous and organic layers were then collected, and the procedure was repeated using halved volumes on the residual sample containing the precipitated protein to maximize the recovery. Collected layers from two rounds of extraction were combined, and the aqueous layer was dried overnight in vacuum centrifuge (Eppendorf), while the lipid fraction was dried under nitrogen gas flow. Dried samples were stored at −80°C.

Targeted metabolite analysis was performed with reverse- and normal-phase separations on a Thermo Fisher Scientific ultra-HPLC (UHPLC)^+^ series coupled with a TSQ Quantiva mass spectrometer operated in positive and negative ion mode with switching mode, as previously described (*88*). Stable isotope-labeled standards were used to allow quantification of the detected metabolites, and data were normalized to cell count.

### Fitness effect of phosphorylation sites and MYC target sites by CGE assay

CGE assay was performed as described in Pihlajamaa *et al.* (*41*). Briefly, 200,000 to 400,000 early-passage HAP1 cells were transfected with a ribonucleoprotein complex containing tracrRNA and crRNA (250 to 500 ng) with S.p. HiFi Cas9-protein (1 to 2 μg; Integrated DNA Technologies), together with ssODN homology directed repair (HDR) template with a final concentration of 3 nM using CRISPRMAX (Life Technologies) following the manufacturer’s recommendations. For prime editor experiments, plasmids for prime editor and Prime Editing Guide RNA (pegRNA), pCMV-PE2 and pU6-pegRNA-GG-acceptor (#132775 and #132777, respectively, Addgene) (*89*), were transfected using FuGENE HD (Promega) according to the manufacturer’s instructions and 4:1 FuGENE HD:DNA ratio. Half of the cells were collected for genomic DNA (gDNA) isolation on day 2 after transfection, and the other half cultured until day 8 for late time-point sample. Isolation of gDNA, treatment with ribonuclease A, exonucleases I and VII, two-step polymerase chain reaction amplification, sequencing, and data analysis were performed as described in Pihlajamaa *et al.* (*41*). A read count cutoff of 5 to 50 was used for day 2 samples, depending on the sequencing depth. Sequences of crRNAs, HDR donor templates, pegRNAs, and target-specific primers are listed in table S5.

### Gene regulatory network construction

The human gene and paralogous gene pair annotations were downloaded from Ensembl version 54. The paralogous gene pairs were merged into paralog groups one by one, making sure that the groups remained consistent, i.e., all the paralogs of a gene were in one group.

The gene regulatory network has five classes of nodes: TFs, ChIP-seq peaks, cancer-associated SNPs, target genes, and paralog groups. Edges were drawn from each TF node to all its peak nodes and from a peak and SNP nodes to a target gene node if they are within 500 kb of the gene. Target gene nodes were connected to the corresponding paralog group nodes. Features such as ChIP-seq cancer cell lines, peak heights, GWAS traits, SNP *P* values, and the distances of these features from the TSS of a gene (measured as the number of genes that have a TSS closer to the feature), were assigned as attributes to the respective nodes and edges.

The edges between ChIP-seq peak and SNP nodes and the target gene nodes were scored on the basis of two different criteria: the rank of the regulatory feature (peak height or cancer association *P* value) and the rank of its distance from the target gene, both within all features of the same type (peaks in the same cell line or SNPs associated with the same cancer trait). The edge score was then calculated as the fraction of all regulatory relationships of the same type that ranked at least as highly by both criteria as the regulatory relationship being scored.

### Network queries

The regulatory network was searched for matches of short regulatory paths that represented the regulatory relationships of interest. The queried paths ended either to a target gene (GWAS query) or to a paralog group (ChIP-seq query). A path in the regulatory network and a query path match only if there is a mapping (subgraph isomorphism) between their nodes that preserves both node adjacencies and their attribute values (the class of the node and additional constraints such as whether the node represents a gene involved in cell cycle regulation). All network paths matching a query path were searched as follows:

1) Traverse the query path in the reversed direction of the edges starting from the target node (representing the target gene or paralog group) and collect the node at each depth.

2) In the regulatory network, search all the nodes that match the target node of the query.

3) For each matching target node in the regulatory graph, do a depth first search of the network, again in the reverse direction of the edges. The search is continued only if the regulatory network node matches the query node at the same depth. If a matching path is found, then it is added to the match subnetwork.

The target nodes were then ranked using a combined target score calculated as the product of the scores of the incoming edges to the target gene node(s) in the match subnetworks. For each type of edge (from cell line or cancer trait node), only the edge with the smallest score is used in the scoring. The final result of the search is a list of target genes or paralog groups ranked in the ascending order of their total combined scores. The network search algorithm was implemented as a Cytoscape plug-in (*90*). The networks were constructed, and queries were executed in Cytoscape version 2.8.0 using Java version 6.

Permutation testing was used to assess how probable it is to obtain by chance the observed amount of signal from multiple tumor types or cancer traits to a target gene or paralog group. The null hypothesis was that there was no association between the targets and different (cell line or cancer trait) scores that contribute to the combined scores and, thus, the target labels (genes or paralog groups) were exchangeable. The following round of permutations followed by recalculation of scores was performed a hundred thousand times. First, for each cell line or cancer trait, the target node labels (genes or paralog groups) were permuted so that the assignment between the cell line or cancer trait scores and targets was randomized. Then, a combined score was calculated for each target based on the randomized subnetwork. The empirical *P* value was calculated for each target gene or paralog group as the fraction of the rounds in which it got the same or smaller combined score than its combined score in the network query. The reported *P* values were not corrected for multiple hypothesis testing. The results of the queries corresponding to Fig. 1 (C and D) are presented in table S6.

### Modeling drug responses in the single-cell transcriptomics data

Drug effects on gene expression were analyzed using a multivariate regression model with treatment time, drug concentration, cell line identity (parental versus resistant), and cell cycle phase as variables. The model used full factorial design and assumed monotonicity for the time and concentration variables. The expression values were sum normalized for each sample and then ranked over the samples, and the ranks were mapped to [0, 1], zero as the lowest expression value and one as the highest for each gene. A model was fit independently on each gene using the least-squares metric. Specifically, the estimated expression levels β* are

$$\beta^{*}=\text{arg}\;{\text{min}}_{\beta }\;\Sigma_{i,j}{\left(x_{i,j}-\beta_{i,\mathrm{Pj},\mathrm{Dj},\mathrm{Tj},\mathrm{Cj}}\right)}^{2}$$

such that for all *i*

$$\left(\beta_{i,p,d,t,c}\leq \beta_{i,p,d+1,t,c}\;\text{for}\;\text{all}\;p,d,t,\text{and}\;c\;\text{or}\;\beta_{i,p,d,t,c}\geq \beta_{i,p,d+1,t,c}\;\text{for}\;\text{all}\;p,d,t,\text{and}\;c\right)$$ (1)

and

$$\left(\beta_{i\;p,d,t,c}\leq \beta_{i,p,d,t+1,c}\;\text{for}\;\text{all}\;p,d,t,\text{and}\;c\;\text{or}\;\beta_{i,p,d,t,c}\geq \beta_{i,p,d,t+1,c}\;\text{for}\;\text{all}\;p,d,t,\text{and}\;c\right)$$

where *x*_*i*,*j*_ is the normalized expression level of the *i*th gene in the *j*th cell; β_*i*,*p*,*d*,*t*,*c*_ represents the estimated expression level (model) of the *i*th gene for the *p*th cell line, *d*th drug concentration, *t*th time point, and *c*th cell cycle phase; and *P_j_*, *D_j_*, *T_j_*, and *C_j_* are the cell line, drug concentration, time point, and cell cycle phase groups of the *j*th cell, respectively. The problem can be formulated as quadratic programs and were solved with the interior point solver of MATLAB R2019b with default parameters.

Because cell cycle distribution is not independent of treatment time and drug concentration effects, including cell cycle phases estimated with Seurat (*91*) overcorrected the model. To regress out common cell cycle phase effect, while still retaining the early responses to the drugs, cell cycle phase estimation for each cell line was combined with the above model on specific cell cycle phase marker genes (*92*). Specifically, the unknown cell cycle phases *C_j_* in the above model can be solved with the following iterative algorithm: (i) Given current *C_j_*, solve Eq. 1 for the model effects **β** in the relevant genes *i* with the additional constraint of expression being high in specific genes.

Such that for all *c*, *i* where the gene *i* is specific to the cell cycle phase *c*

$$\beta_{i,p,d,t,c\prime }\leq \beta_{i,p,d,t,c}\;\text{for}\;\text{all}\;p,d,t,\text{and}\;c^{\prime }$$ (2)

(ii) Given the current model effects **β**, choose a new set of the most likely cell cycle phases C_j_* for each cell using

$${C_{j}}^{*}=\text{arg}\;{\text{min}}_{\mathrm{Cj}}\;\Sigma_{i}{\left(x_{i,j}-\beta_{i,\mathrm{Pj},\mathrm{Dj},\mathrm{Tj},\mathrm{Cj}}\right)}^{2}$$ (3)

Iterating (i) and (ii) converges into an optimum of Eq. 1 subject to Eq. 2 with respect to both **β** and *C_j_* free. The initial cell cycle phases *C_j_* were drawn independently from a uniform distribution, and a total of 100 iterations with 10 restarts were used. The cell cycle phases derived using this procedure were then used as regressors in the model of Eq. 1 in estimating the drug effects for all genes.

The contribution of each variable to the model was assessed by dropping the relevant variable from the model and performing rank variance analysis. Genes were then ranked on the basis of the explanatory power of drug effect in the drug resistant versus the sensitive parental cell line, and the top-ranked genes from each cell line pair were used in the subsequent analyses.

### MYC target genes

#### RNA-seq and ChIP-seq for MYC target gene analysis

Wild-type (RRID:CVCL_0492) and Myc-null Rat1 fibroblasts (*74*) were a gift from J. Sedivy, Brown University, and R. Bernards, Netherlands Cancer Institute. The cells were maintained in DMEM with 10% FBS and antibiotics. To study the Myc targets in rodent cells, different levels of MYC were ectopically expressed in Myc-null Rat1 cells using a lentiviral construct for the human MYC at different values of multiplicity of infection [MOI = 1 and MOI = 3; construct and transduction protocol as described in Sahu *et al.* (*93*)]. RNA-seq was performed from wild-type and Myc-null Rat1 cells as well as from Myc-null Rat1 with MYC transduction (MOI = 1 from two independent experiments and MOI = 3 from one experiment). Total RNA from four replicates for each condition was isolated using RNeasy Mini kit (QIAGEN), and libraries were generated using KAPA stranded mRNA-seq kit for Illumina (Roche) and sequenced using HiSeq 4000 (Illumina). The reads were aligned to rn6 using tophat2 (v2.0.13) (*94*), and differentially expressed genes between Rat1 Myc-null cells and each of the MYC-expressing condition were analyzed using cuffdiff (v2.2.1) (*95*) with default parameters for first-strand library type. ChIP was performed using anti-MYC antibody (#06-340, Millipore, RRID:AB_11214006; 5 μg per reaction) and normal rabbit IgG as previously described (*96*). ChIP-seq libraries were prepared using NEBNext Ultra II DNA Library Prep kit (New England Biolabs) and sequenced on HiSeq 4000. The reads were aligned to rn6 using bowtie2 (version 2.2.4) (*97*), and peaks were called using MACS2 (version 2.1.1) (*98*), with default narrow peak parameters against normal IgG.

For human cells, the following previously published datasets were used: ChIP-nexus data for colon cancer cell lines GP5d (RRID:CVCL_1235), LoVo, and COLO320DM (RRID:CVCL_0219) from Palin *et al.* (*99*) (EGAD00001004099) and Assay for Transposase-Accessible Chromatin using sequencing (ATAC-seq) data for GP5d cells from Sahu *et al.* (*93*) (GSE180158). In the genome browser snapshots, the traces from BAM coverage files are shown, and in MYC target gene analysis, the peaks called for GP5d and LoVo as reported in Palin *et al.* (*99*) were used. For gene expression profiling, RNA-seq data from GP5d cells treated with either siRNA against MYC or nontarget control from Palin *et al.* (*99*) (EGAD00001004098) were reanalyzed by aligning the reads to hg19 using tophat2 (v2.0.13) (*94*) and by performing differential expression analysis using cuffdiff (v2.2.1) (*95*), with default parameters for first-strand library type.

#### Identifying high-confidence MYC target genes conserved between human and rodent cells

To identify putative MYC targets in human colon cancer cells, different datasets were integrated in a stepwise manner. First, MYC ChIP-nexus peaks from GP5d and LoVo cells were compared, and only the peaks common to both cell lines were used in the analysis. Second, the genes expressed in GP5d cells were identified on the basis of the RNA-seq data [fragments per kilobase of exon per million mapped reads (FPKM) > 2 in control cells], and of these, 7254 genes with MYC ChIP-nexus peaks within ±1000 base pairs (bp) of their transcription start site were taken for further analysis. On the basis of differential expression analysis between MYC-siRNA and nontargeting control, the 1534 genes of 7254 with an FDR of <0.1 and a fold change of >1.3 or <−1.3 were extracted. In addition, the 430 genes of 7254 with a difference larger than 50 units in FPKM values between MYC-siRNA and nontargeting conditions were extracted. Last, merging the lists of 1534 and 430 differentially expressed genes resulted in 1689 MYC target genes in human colon cancer cells.

Similarly, for the Myc targets in rat cells, first, the genes expressed in Rat1 wild-type cells (FPKM > 2) having an MYC ChIP-seq peak in Rat1 Myc-null cells with an MYC MOI of 3 within ±1000 bp of their transcription start site were identified. Of these 6552 genes, the 1644 genes with an FDR of <0.1 and a fold change of >1.3 or <−1.3 under at least three of four conditions (Rat1 wild-type versus Rat1 Myc-null; MYC MOI = 1 from experiment 1 versus Myc-null; MYC MOI = 1 from experiment 2 versus Myc-null; and MYC MOI = 3 versus Myc-null) were extracted. In addition, the 513 genes of 6552 with a difference larger than 50 units in their FPKM values under at least two of four above conditions were extracted. Last, merging the lists of 1644 and 513 differentially expressed genes resulted in 1911 MYC target genes in rat fibroblast cells. Last, overlap analysis of the putative MYC targets in human and rat cells resulted in 490 common high-confidence MYC targets.

### MS analyses

#### Cell line phosphoproteomics

The LC-ESI-MS/MS analyses of phosphoenriched and nonenriched multiplexed samples were performed on the Orbitrap Fusion Lumos coupled to a nanoLC Dionex Ultimate 3000 UHPLC with a 2-cm trap column (Thermo Fisher Scientific) and a 50-cm C18 analytical column (75-μm inner diameter, 5 μm, 100 Å; Acclaim PepMap) using 120- or 180-min separations.

Mass spectra were acquired in positive ion mode using SPS-MS^3^ acquisition mode as reported previously (*100*). Mass spectra were acquired in a mass/charge ratio (*m*/*z*) range of 375 to 1500 with a resolution of 120,000. The most intense ions were selected for collision-induced dissociation–MS2 fragmentation in the ion trap with 35% normalized collision energy. Coselected precursors for SPS-MS^3^ underwent higher-energy collision dissociation (HCD) fragmentation with 65% normalized collision energy and were analyzed in the Orbitrap as described by Navarrete-Perea *et al.* (*101*).

The raw data were searched with Proteome Discoverer v.2.3 (Thermo Fisher Scientific) using Mascot algorithm against UniProt 2019_02 release using *Homo sapiens* taxonomy filter. Up to two missed cleavages were allowed. Carbamidomethyl (C), and TMTpro or TMT6plex on (K) and (N-term) were used as static modifications. Oxidation (M), acetyl (protein N-term), phospho (Y), and phospho (ST) were used as variable modifications. Percolator was used for FDR estimation, and only peptide identifications of high confidence (FDR < 1%) were included in the analyses.

#### Proteome integral solubility alteration

PISA samples were analyzed on a Thermo Orbitrap Q-Exactive HF, equipped with an EASY-Spray source and connected to an UltiMate 3000 RSLC nanoUPLC system (Thermo Fisher Scientific). Peptide separation was performed using an EASY-Spray C18 reversed-phase nanoLC column (Acclaim PepMap RSLC; length, 50 cm; inner diameter, 2 75 μm; particle size, 2 μm; pore size, 100 Å; Thermo Fisher Scientific) at 55°C and a flow rate of 300 nl/min. Peptides were separated using a binary solvent system consisting of 0.1% (v/v) formic acid (FA) and 2% (v/v) ACN (solvent A) and 98% ACN (v/v) and 0.1% (v/v) FA (solvent B).

Mass spectra were acquired in an *m*/*z* range of 375 to 1500 with a resolution of 120,000 at *m*/*z* 200. Automatic gain control target was set to 3 × 10^6^ with a maximum injection time of 100 ms. The 17 most abundant peptide ions were selected for HCD with normalized collision energy value set at 33. The ion abundance threshold was set at 0.1% with charge exclusion of *z* = 1 ion. The MS/MS spectra were acquired at a resolution of 60,000, with a target value of 2 × 10^5^ ions and a maximum injection time of 120 ms.

Protein identification and quantification were performed using MaxQuant search engine and the UniProt human proteome reference database (UP000005640) for matching MS/MS spectra and TMT-base quantification. Cysteine carbamidomethylation was used as a fixed modification; methionine oxidation, arginine, and glutamine deamination were used as variable modifications for both identification and quantification. Trypsin cleavage with maximum two missed cleavages were allowed, and only high confidence (1% FDR) was retained in the dataset at both protein and peptide levels. After removing contaminants, only proteins with at least two unique peptides were included in the final dataset.

#### Polar metabolite analysis

Analyses were conducted as in McKenna *et al.* (*88*). Briefly, a Thermo Fisher Scientific UHPLC^+^ series coupled with a TSQ Quantiva mass spectrometer (Thermo Fisher Scientific, Waltham, MA, USA) was used with an ESI source, operated in positive and negative ion modes at the same time. The electrospray voltage was set to 3500 V for the positive ionization and to 2500 V for the negative ionization. Nitrogen at 48 mtorr and 420°C was used as a drying gas for solvent evaporation.

For reversed-phase analysis, samples were reconstituted in 0.1 ml of a 10 mM ammonium acetate water solution containing a mixture of eight internal standards at the concentration of 10 μM (proline, valine D8, leucine D10, lysine U13, glutamic acid C13, phenylalanine D5, succinic acid D3, and serotonin D4). Samples were analyzed with an ACE Excel 2 C18 PFP (100 A, 150 × 2.1 mm, 5 μm) column. The column was conditioned at 30°C. The mobile phase consisted of (A) a 0.1% of FA water solution and (B) a 0.1% of FA ACN solution. The mobile phase was pumped at a flow rate of 500 μl/min programmed as follows: initially held at 100% A for 1.60 min and then subjected to a linear decrease from 100 to 70% A in 2.4 min and to 10% in 0.5 min, then held constant for 0.5 min, and brought back to initial condition after 0.1 min. The Xcalibur software (Thermo Fisher Scientific, Waltham, MA, USA) was used for data acquisition. Putative recognition of all detected metabolites was performed using a selected reaction monitoring (SRM) MS analysis.

For normal-phase analyses, samples were reconstituted in 0.1 ml of ACN:10 mM ammonium carbonate water solution (7:3, v/v) containing a mixture of three internal standards at the concentration of 10 μM (glutamic acid C13, succinic acid D3, and AMP). The samples were analyzed with a Bridged Ethylene Hybrid (BEH) amide (150 × 2.1 mm, 1.7 μm) column. The column was conditioned at 30°C. The mobile phase consisted of (A) a 0.1% of ammonium carbonate water solution and (B) an ACN solution. The mobile phase was pumped at a flow rate of 600 μl/min programmed as follows: initially stayed at 20% A for 1.50 min, then subjected to a linear increase from 20 to 60% A in 2.5 min, kept at this percentage for 1 min, and then brought back to initial condition after 0.1 min. The Xcalibur software (Thermo Fisher Scientific, Waltham, MA, USA) was used for data acquisition. Putative recognition of all detected metabolites was performed using an SRM MS analysis.

#### Invasive front tumor samples

The LC-ESI-MS/MS analyses of phosphoenriched and nonenriched multiplexed samples were performed on a nanoflow HPLC system (Easy-nLC1200, Thermo Fisher Scientific) coupled to the Orbitrap Fusion Lumos mass spectrometer (Thermo Fisher Scientific, Bremen, Germany) equipped with a nanoelectrospray ionization source and FAIMS interface (Thermo Fisher Scientific). Three FAIMS compensation voltages, −40, −60, and − 80 V, were used. Online chromatography was performed with one of the two different column setups: (i) Samples first loaded on a trapping column and subsequently separated inline on a 15-cm C18 column (75 μm by 15 cm; ReproSil-Pur 3 μm 120 Å C18-AQ, Dr. Maisch HPLC GmbH, Ammerbuch-Entringen, Germany); (ii) samples were loaded on an in-house packed 25-cm, 75-μm–inner diameter capillary column with 1.9-μm Reprosil-Pur C18 beads (Dr. Maisch, Ammerbuch, Germany) with column temperature maintained at 60°C. The mobile phase consisted of water with 0.1% FA (solvent A) or ACN/water (80:20, v/v) with 0.1% FA (solvent B). A 120-min two-step gradient from 7 to 24% of eluent B in 62 min to 39% of eluent B in 48 min, followed by a wash stage with 100% of eluent B, was used to eluate peptides.

MS data were acquired automatically by using Thermo Xcalibur 4.4 software (Thermo Fisher Scientific). A data-dependent acquisition method consisted of an Orbitrap MS survey scan with a mass range of 350 to 1750 *m*/*z*, with a resolution of 120,000, followed by HCD fragmentation for the most intense peptide ions in a top speed mode with a cycle time of 1 s for each compensation voltage. MS/MS spectra were collected with resolution of 50,000.

Data files were searched using ProteomeDiscoverer 2.5 software (Thermo Fisher Scientific) connected to an in-house server running the Mascot 2.7.0 software (Matrix Science). Data were searched against a SwissProt (version 2021_4) database using *H. sapiens* taxonomy filter. Carbamidomethyl (C), TMTpro (K), and TMTpro (N-term) were used as static modifications. Oxidation (M), acetyl (protein N-term), phospho (Y), and phospho (ST) were used as variable modifications. Abundance values for peptides and proteins were calculated on the basis of intensities of TMTpro reporter ions. Only unique peptides were used for the protein level quantitation, and only peptide identifications of high confidence (FDR < 1%) were included in the analyses.

### Quantification and statistical analyses

FDR of peptide identifications was estimated using target-decoy approach, and peptides with FDR <1% were included in the analyses. Statistical analyses of the proteomics data were performed using R software (version 4.1). All the *t* tests, Wilcoxon signed-rank tests, and Fisher’s exact tests were two sided. Benjamini-Hochberg adjustment was used to account for multiple hypothesis testing in the functional enrichment analyses. All the measurements were taken from distinct samples. Statistical details of experiments can be found in the figure legends. Significance testing for gene regulatory network queries and modeling of the drug responses in the single-cell transcriptomics data are described under relevant sections in Materials and Methods. A priori sample size calculations were not performed. Sample sizes for the analyses were determined by the nature of the data (such as the number of genes, proteins, or phosphorylation sites belonging to a certain biological category or the number of edited lineages in the CGE assay).
