# Supplementary material for: Diverse oncogenes use common mechanisms to drive growth of major forms of human cancer
Source: Sci Adv. 2025 Aug 20;11(34):eadt1798. doi: 10.1126/sciadv.adt1798 (PMC13155486; doi:10.1126/sciadv.adt1798)
Supplement: Supplementary file 1 — Figs. S1 to S8 Tables S1 to S6 [file sciadv.adt1798_sm.pdf]

Science Advances
AAAS

Supplementary Materials for

Diverse oncogenes use common mechanisms to drive growth of major forms of human cancer

Otto Kauko *et al.*

Corresponding author: Jussi Taipale, jt37@sanger.ac.uk

Sci. Adv. **11**, eadt1798 (2025)
DOI: 10.1126/sciadv.adt1798

This PDF file includes:

Figs. S1 to S8
Tables S1 to S6

A

Cancer Type	Cell line	TF	Number of peaks (p<0.05)	Motif	E-value of motif	Source
Colon carcinoma	GP5d	CTNNB1	8128		$2.7x10^{-41}$	this work
Colon carcinoma	LoVo	TCF7L2	1596		$6.0x10^{-58}$	Tuupanen et al. 2009
Breast carcinoma	MCF-7	ESR1	5863		$4.5x10^{-112}$	this work
Prostate carcinoma	VCaP	AR	32479		$4.4x10^{-59}$	Wei et al. 2010
Prostate carcinoma	VCaP	ERG	14891		$7.4x10^{-36}$	Wei et al. 2010
Ewing's sarcoma	SK-N-M(	FLI1	3501		$2.2x10^{-848}$	Wei et al. 2010
Rhabdomyosarcoma	CRL-206	PAX3	7476		$1.9x10^{-13}$	this work
Rhabdomyosarcoma	CRL-206	GLI1	51386		$1.0x10^{-80}$	this work

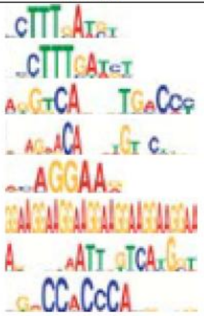

B

	Cell line	Target change	Down	Up
**ESR1**	MCF-7	Not applicable	727	856
**AR**	VCaP	Not applicable	1121	1125
**ERG**	VCaP		105	64
**CTNNB1**	GP5D		2872	2431
**EWS/FLI1**	SK-N-SC		1616	1501
**GLI1**	CRL-2061		392	377
**PAX3**	CRL-2061		1408	1370

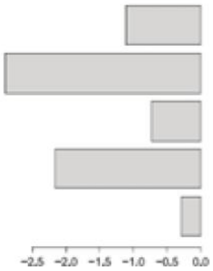

Wei et al. 2010

Fig. S1. Transcriptional regulators analyzed using ChIP sequencing.

(A), Cell lines used in the experiment, the number of peaks identified for each factor, and PWM models for the TF binding sites within ChIP-seq peaks (*75*, *76*).

(B), Regulation of target gene expression following RNAi of the TF. Not applicable = instead of RNAi, these TFs were activated by their natural ligand (*76*).

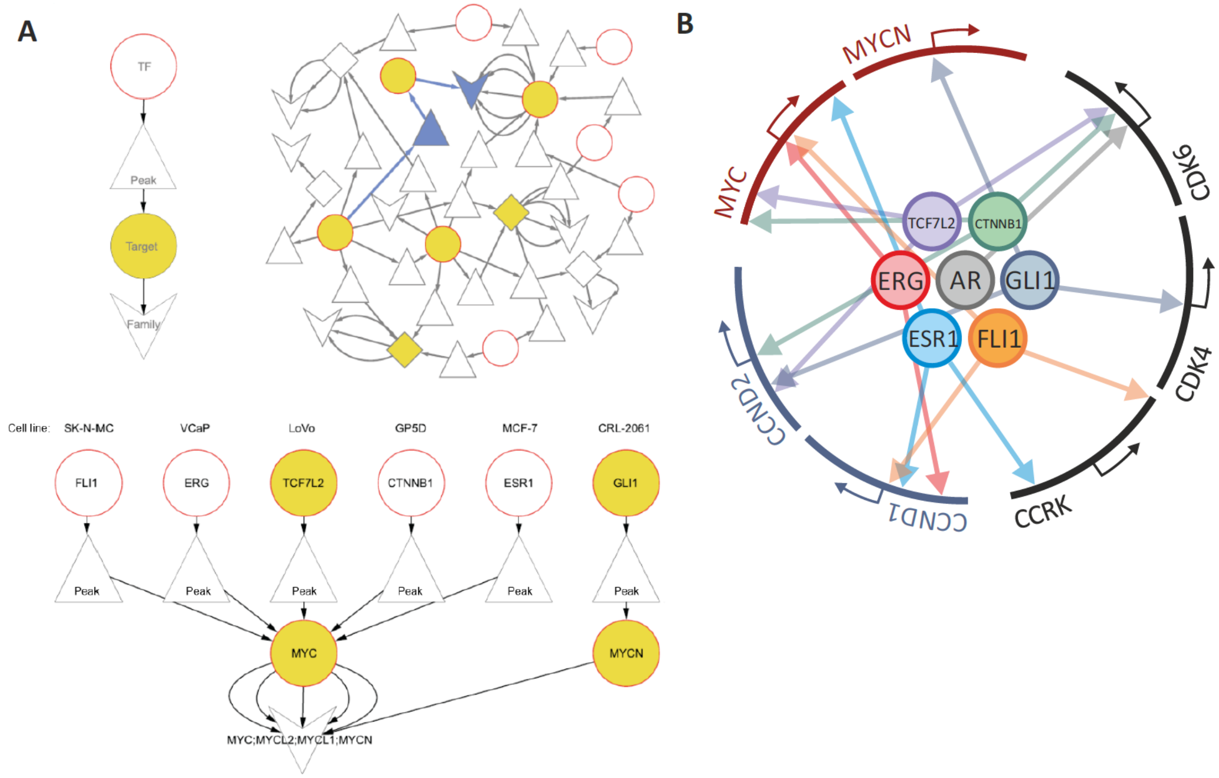

Fig. S2. Gene regulatory network queries.

(A), The networks containing all the gene regulatory data from ChIP-seq experiments and GWAS analysis are very large. Therefore, in order to identify common targets of the transcription factors, we implemented a subgraph isomorphism algorithm that searches for paths converging on a single gene or paralog group. Searched path for one cell line is illustrated in top left panel, and overlaid on an example network, in top right panel. Paths converging on the MYC paralog group in multiple cell lines are shown in the bottom panel as an example.

(B), Results of the ChIP-seq analysis shown for the enhancers of selected paralog groups. TF Binding site positions are indicated with an arrow with respect to transcription start site (TSS). Red: MYC genes, Black: CDK4/6, CCRK. Blue: D-type cyclins.

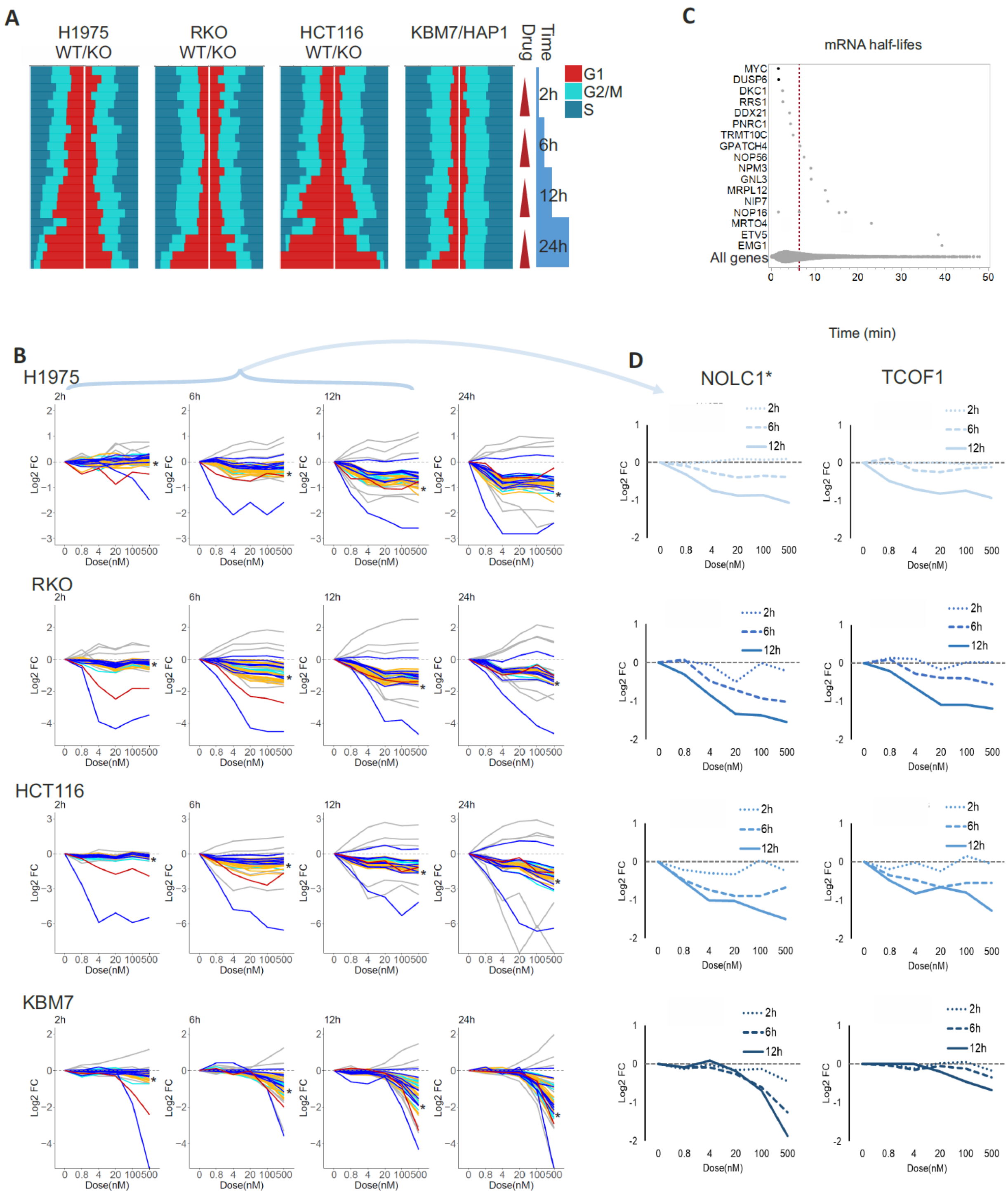
A
H1975 WT/KO
RKO WT/KO
HCT116 WT/KO
KBM7/HAP1
Drug
Time
2h
6h
12h
24h
G1
G2/M
S
C
mRNA half-lifes
MYC
DUSP6
DKC1
RRS1
DDX21
PNRC1
TRMT10C
GPATCH4
NOP56
NPM3
GNL3
MRPL12
NIP7
NOP16
MRTO4
ETV5
EMG1
All genes
0 10 20 30 40 50
Time (min)
B
H1975
RKO
HCT116
KBM7
2h
6h
12h
24h
Log2 FC
Dose(nM)
0 0.8 4 20 100500
D
NOLC1*
TCOF1
2h
6h
12h
Log2 FC
Dose(nM)
0 0.8 4 20 100 500

Fig. S3. Common transcriptional mechanisms activated by phosphorylation signaling

(A), Cell cycle distributions of the cell line pairs. Red: G1, Dark blue: S, Cyan: G2/M. G1 arrest occurs predominantly in the sensitive version of the cell line and at lower drug concentrations.

(B), Expression changes in the common target genes as a function of drug concentration at 2h, 6h, 12h, and 24h time points. Intersection of the Top ranked 500 genes is shown for each parental (drug sensitive) cell line for clarity. Red=MYC, Dark blue= MYC target genes, Cyan = Ribosome biogenesis (GO:0042254), Yellow = MYC target involved in ribosome biogenesis, * = NOLC1.

(C), mRNA half-lives (33) of the common target genes in Fig 2H. Median of all genes is shown with a red dashed line.

(D), Expression of NOLC1 and TCOF1 as a function of drug concentration at 2h, 6h, and 12h timepoints in the parental (drug sensitive) cell lines.

The data were generated from NCI-H1975, RKO, HCT116, and KBM7/HAP1 cells as presented **table S2**.

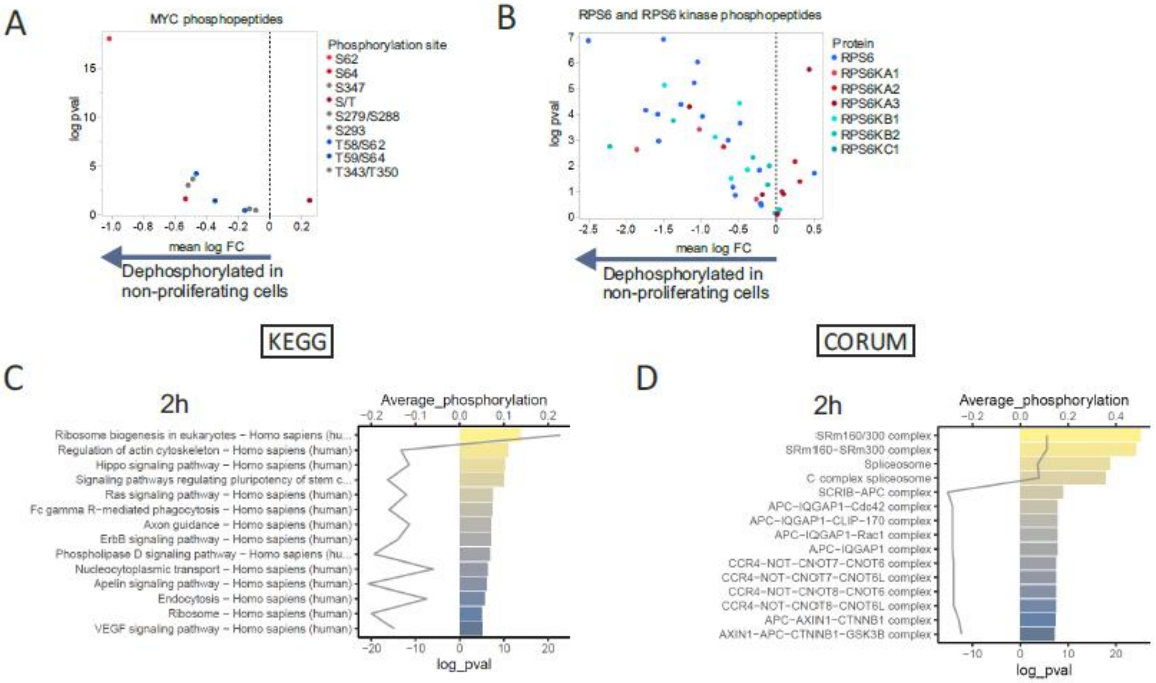

Fig. S4. Phosphoproteomics profiling of shared targets of growth regulatory pathways

(A), Downregulation of MYC phosphopeptides in non-proliferating cells.

(B), Downregulation of RPS6 and p70 S6K phosphopeptides in non-proliferating cells.

(C), Top differentially phosphorylated KEGG pathways by p-value at 2h time point are indicated in the bar graph. Fold changes are shown with a line. Data and bar graph colors correspond to the volcano plot in **Fig 2B**.

(D), Top differentially phosphorylated CORUM database complexes by p-value at 2h time point are indicated in the bar graph. Fold changes are shown with a line. Data and bar graph colors correspond to the volcano plot in **Fig 2C**.

The data were generated from NCI-H1975, RKO, HCT116, LoVo (both resistance mechanisms), MCF-7 (Temsirolimus, both resistance mechanisms), and KBM7/HAP1 (both drugs) cell lines as presented in **table S2**. p-values were calculated using two-tailed one sample t-test.

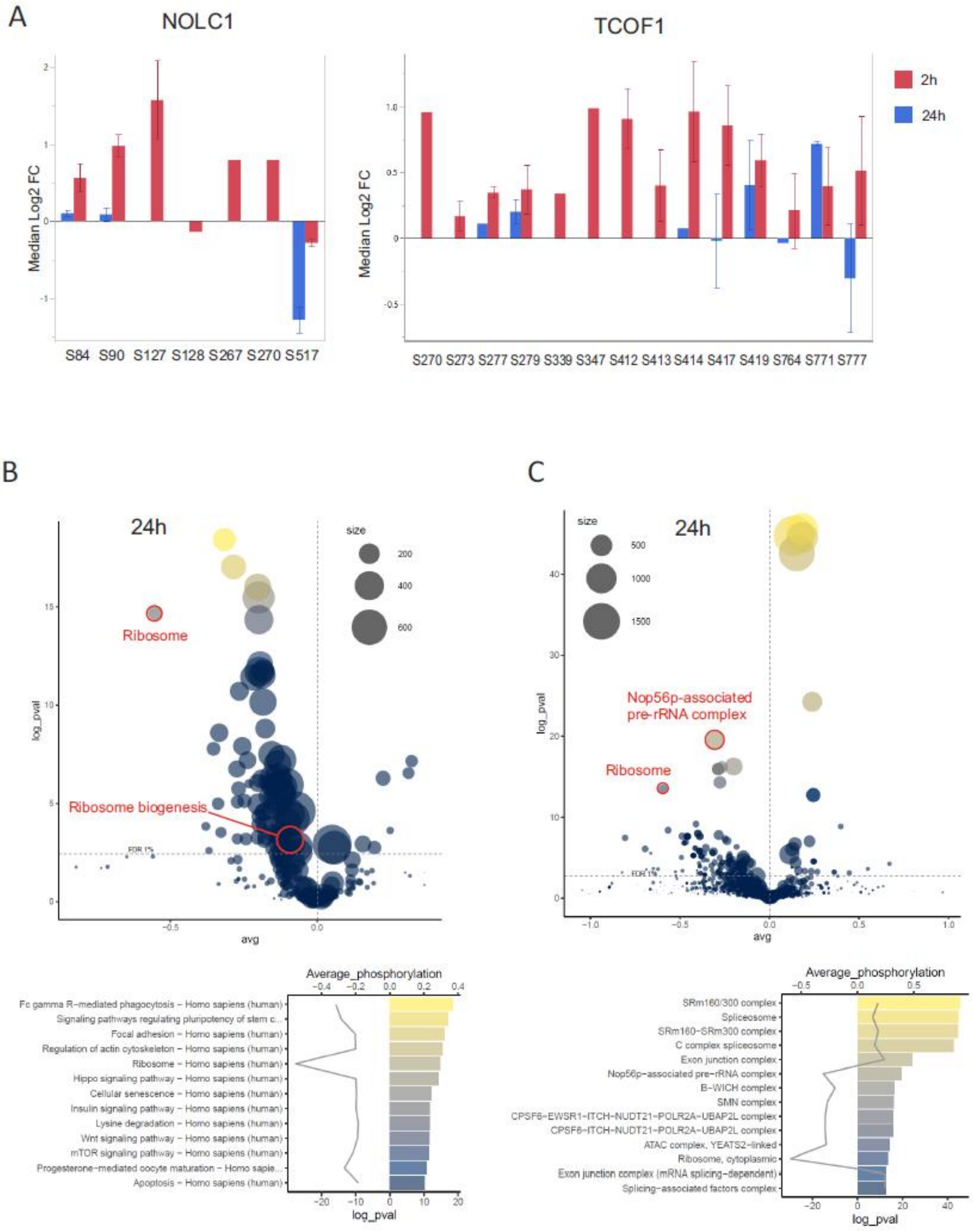
A
NOLC1
Median Log2 FC
S84 S90 S127 S128 S267 S270 S517
TCOF1
Median Log2 FC
S270 S273 S277 S279 S339 S347 S412 S413 S414 S417 S419 S764 S771 S777
2h
24h
B
24h
size
200
400
600
Ribosome
Ribosome biogenesis
FDR 1%
log_pval
avg
Average_phosphorylation
Fc gamma R-mediated phagocytosis - Homo sapiens (human)
Signaling pathways regulating pluripotency of stem c...
Focal adhesion - Homo sapiens (human)
Regulation of actin cytoskeleton - Homo sapiens (human)
Ribosome - Homo sapiens (human)
Hippo signaling pathway - Homo sapiens (human)
Cellular senescence - Homo sapiens (human)
Insulin signaling pathway - Homo sapiens (human)
Lysine degradation - Homo sapiens (human)
Wnt signaling pathway - Homo sapiens (human)
mTOR signaling pathway - Homo sapiens (human)
Progesterone-mediated oocyte maturation - Homo sapie...
Apoptosis - Homo sapiens (human)
log_pval
C
24h
size
500
1000
1500
Nop56p-associated
pre-rRNA complex
Ribosome
FDR 1%
log_pval
avg
Average_phosphorylation
SRm160/300 complex
Spliceosome
SRm160-SRm300 complex
C complex spliceosome
Exon junction complex
Nop56p-associated pre-rRNA complex
B-WICH complex
SMN complex
CPSF6-EWSR1-ITCH-NUDT21-POLR2A-UBAP2L complex
CPSF6-ITCH-NUDT21-POLR2A-UBAP2L complex
ATAC complex, YEATS2-linked
Ribosome, cytoplasmic
Exon junction complex (mRNA splicing-dependent)
Splicing-associated factors complex
log_pval

Fig. S5. Phosphoproteomics profiling of shared targets of growth regulatory pathways

(A), Phosphorylation changes in NOLC1 and TCOF1 sites previously reported to undergo pyrophosphorylation.

(B), KEGG pathway phosphorylation changes at 24 hour time point. Volcano plot: Average differential phosphorylation between proliferating and non-proliferating cells in all KEGG pathways excluding the KEGG DISEASE category is shown as log fold change on X-axis. p-value for one sample t-test is shown on Y-axis. Size of the circles indicates the number of phosphopeptides identified in the KEGG pathway. Bar graph: Top pathways by p-value are indicated in the bar graph with colors corresponding to the volcano plot. Fold changes are shown with a line.

(C), Protein complex phosphorylation changes at 24 hour time point Volcano plot: Average differential phosphorylation between proliferating and non-proliferating cells in all CORUM database protein complexes is shown as log fold change on X-axis. p-value for one sample t-test is shown on Y-axis. Size of the circles indicates the number of phosphopeptides identified in protein complexes. Bar graph: Top complexes by p-value are indicated in the bar graph with colors corresponding to the volcano plot. Fold changes are shown with a line.

The data were generated from NCI-H1975, RKO, HCT116, LoVo (both resistance mechanisms), MCF-7 (Temsirolimus, both resistance mechanisms), and KBM7/HAP1 (both drugs) cell lines as presented in Table EV1. p-values were calculated using two-tailed one sample t-test. Dashed line in panels C and D indicates 1% Benjamini-Hochberg FDR.

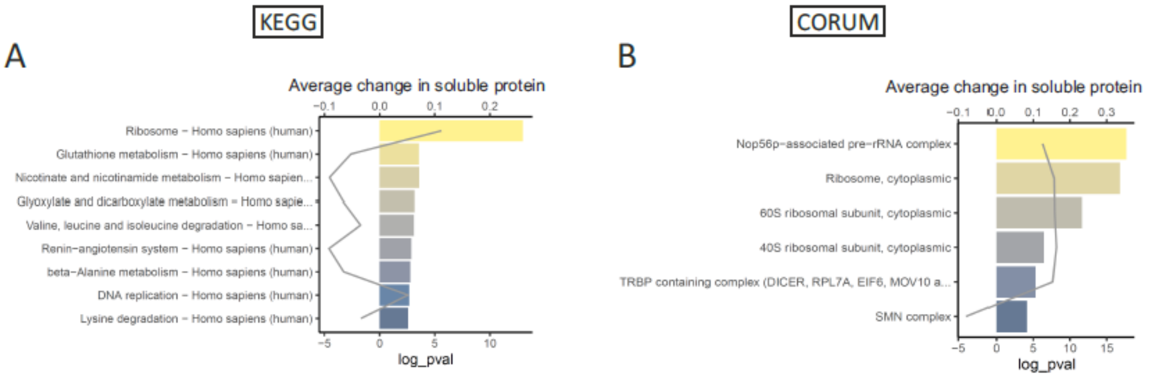

Fig. S6. Protein interaction changes in shared targets of growth regulatory pathways

(A), Top KEGG pathway protein stability changes by p-value at 2h time point are indicated in the bar graph. Fold changes are shown with a line. Data and bar graph colors correspond to the volcano plot in **Fig. 3B**.

(B), Top protein stability changes in CORUM data base complexes by p-value at 2h time point are indicated in the bar graph. Fold changes are shown with a line. Data and bar graph colors correspond to the volcano plot in **Fig. 3C**.

p-values were calculated using two-tailed one sample t-test. Data were generated from NCI-H1975, RKO, and HCT116 cell line pairs, as presented in **table S2**, in 5 replicates for each condition. For each cell line pair, thermal stability change was calculated using the median of these replicates. The t-tests were then calculated for the means of thermal stability changes across all cell line pairs.

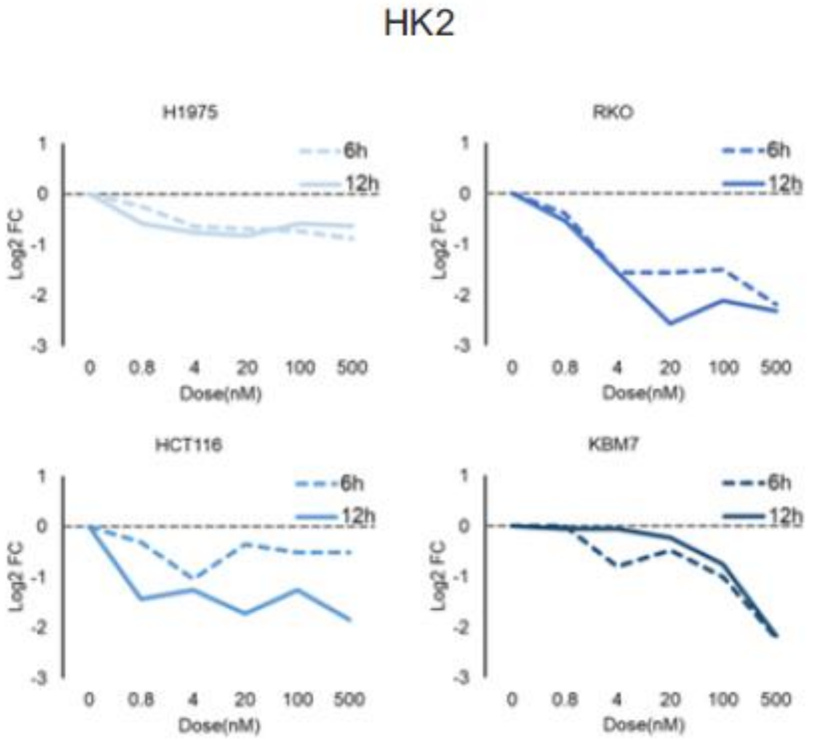

Fig. S7. HK2 mRNA expression in response to treatment with cytostatic drugs.

Expression of HK2 as a function of drug concentration at 6h and 12h timepoints in the parental (drug sensitive) cell lines. NCI-H1975 cells were treated with osimertinib, RKO and HCT116 cells with trametinib, and KBM-7 cells with imatinib.

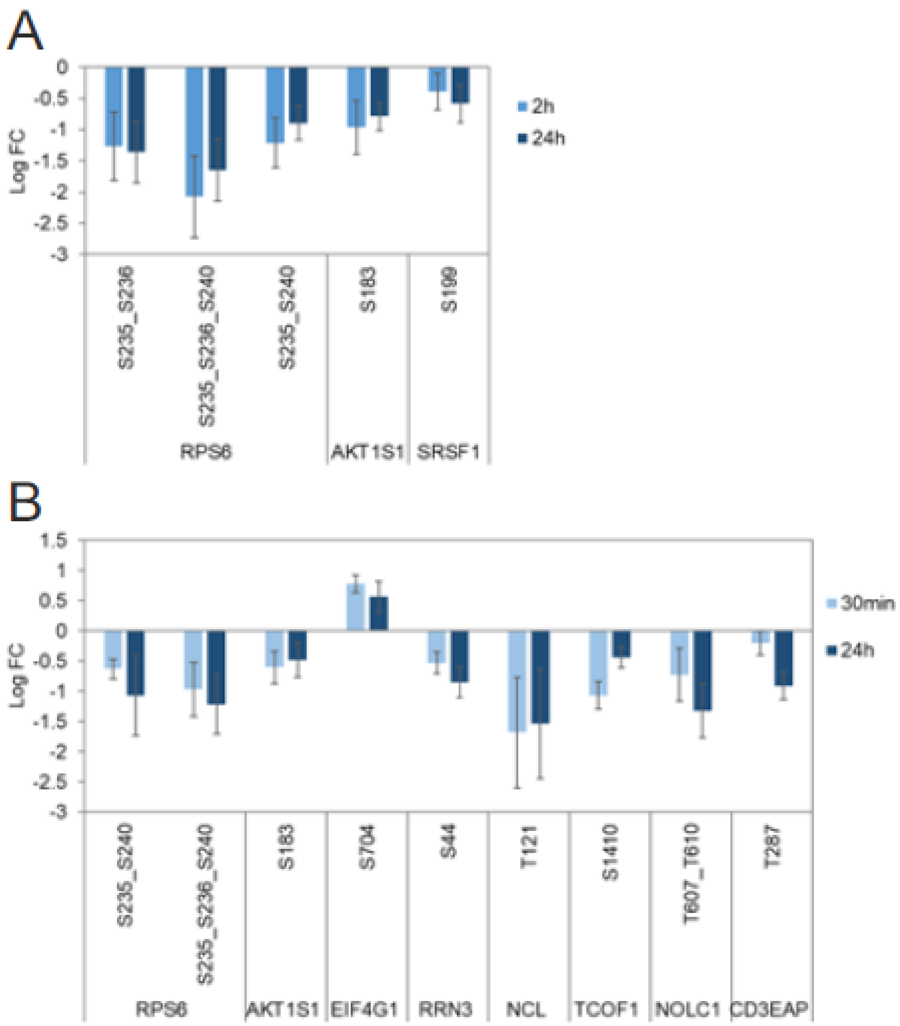

Fig. S8. Phosphorylation sites selected for validation with CGE assay.

(A), Experiment one (2h and 24h timepoints): NCI-H1975, RKO, HCT116, LoVo (both resistance mechanisms), MCF-7 (Temsirolimus, both resistance mechanisms), and KBM7/HAP1 (both drugs) cell lines.

(B), Experiment two (30min and 24h timepoints): NCI-H1975, RKO, MCF-7 (Temsirolimus, NF1 knockout), KBM7/HAP1 (both drugs), A549, T47D, and BT474 cell lines.

The bar graphs represent mean values ± S.E.M for the log2 FC calculated between proliferating and non-proliferating cells in each cell line pair. Negative values indicate downregulation with cytostatic drug.

Table S1. Cell lines

Selected cell lines, driver mutations and represented cancer types

Cell line	Cancer	Mutation	Consequence	Percentage of human tumors affected	PMID
Gp5D	Colon	APC loss	Wnt pathway activation	90	11057903
Gp5D	Colon	KRAS 12D	MAPK pathway activation	56	31589789
LoVo	Colon	APC loss	Wnt pathway activation	90	11057903
LoVo	Colon	KRAS G13D	MAPK pathway activation	56	31589789
MCF-7	Breast	ER amplification	Estrogen signaling pathway activation	70	33369357
MCF-7	Breast	PIK3CA E545K	PIK3CA/AKT/mTOR pathway activation	77	39322687
VCaP	Prostate	AR amplification	Androgen signaling pathway activation	70	33369357
VCaP	Prostate	TMPRSS2-ERG fusion	Activation of ETS signaling	70	18781147
SK-N-MC	Ewing's sarcoma	EWSR1-FLI1 fusion	Activation of ETS signaling and binding to GGAA repeats	100	36505823
CRL-2061	Rhabdomyosarcoma (alveolar subtype)	PAX3-FOXO1 fusion	FOXO1 activation and binding to PAX targets	80	36722003
CRL-2061	Rhabdomyosarcoma	GLI1 amplification	Hh pathway activation	20-50	20818440
HCT116	Colon	KRAS G13D	MAPK pathway activation	56	31589789
RKO	Colon	BRAF V600E	MAPK pathway activation	56	31589789
A549	lung adenocarcinoma	KRAS	MAPK pathway activation	45	31406302
NCI-H1975	lung adenocarcinoma	EGFR L858R	Activation of downstream pathways inlcuding MAPK	20-50	39614090
BT-474	Breast	ERBB2 amplification	Activation of downstream pathways inlcuding MAPK	20	39039196
T47D	Breast	ER amplification	Estrogen signaling pathway activation	70	33369357
T47D	Breast	PIK3CA H1047R	PIK3CA/AKT/mTOR pathway activation	77	39322687
K562	CML	BCR-ABL fusion	Activation of downstream pathways inlcuding JAK/STAT and MAPK	100	35767897
KBM-7	CML	BCR-ABL fusion	Activation of downstream pathways inlcuding JAK/STAT and MAPK	100	35767897

Table S2. Generated drug resistant cell lines

Cell lines, cytostatic kinase inhibitors, and resistance mechanisms in the cell lines generated for this study

**Cancer**	**Cell line**	**Drug**	**Resistance mechanism**
Colon	HCT116	Trametinib	PTEN knockout
Colon	LoVo	Trametinib	PTEN knockout or PIK3CA H1047R
Colon	RKO	Trametinib	PTEN knockout
Lung	A549	Trametinib	PTEN knockout
Lung	NCI-H1975	Osimertinib	PTEN knockout
Breast	BT-474	Lapatinib	PTEN knockout
Breast	T47D	Palbociclib	RB1 knockout
Breast	MCF-7	Palbociclib	RB1 knockout
Breast	MCF-7	Temsirolimus	NF1 Knockout or normal media with 10% serum
CML	K562	Imatinib	NF1 Knockout
CML	KBM-7	Trametinib	HAP1 cell line
CML	KBM-7	Imatinib	HAP1 cell line

Table S3. Phosphorylation sites regulated by cytostatic drugs
Fold changes were calculated by comparing the phosphopeptide abundances between non-proliferating parental cells treated with cytostatic drug) and proliferating cells (parental cells, resistant cells, and resistant cells treated with drug). Table represents sites, whose phosphorylation sites were detected ≥3 in cell line pairs with median Log2 FC of > 0.5 or < -0.5 in both time points.
Negative values indicate downregulation with cytostatic drug.

Experiment one (2h and 24h timepoints): NCI-H1975, RKO, HCT116, LoVo (both resistance mechanisms), MCF-7 (Temsirolimus, both resistance mechanisms), and KBM7/HAP1 (both drugs) cell line pairs

Gene name	Uniprot	Phosphorylation sites	Log2 FC 2h	Log2 FC 24h
ACIN1	Q9UKV3	1xPhospho;[S240]	-0.77	-0.53
ANKRD17	O75179	1xPhospho;[S2401]	-0.67	-0.74
EIF4G1	Q04637	1xPhospho;[S1231]	-0.54	-1.00
MARCKS	P29966	1xPhospho;[S101]	-0.89	-1.12
ZYX	Q15942	1xPhospho;[S259]	-0.59	-0.95
PALLD	Q8WX93	1xPhospho;[S893]	-0.60	-0.71
ATXN2L	Q8WWM7	1xPhospho;[S449]	-0.66	-0.63
SETX	Q7Z333	1xPhospho;[S1663]	-0.55	-0.79
MAP7D1	Q3KQU3	1xPhospho;[S446]	-0.51	-0.71
EIF4G1	Q04637	1xPhospho;[S1231]	-0.62	-0.84
PDS5B	Q9NTI5	1xPhospho;[S1257]	-1.03	-0.56
TXLNA	P40222	1xPhospho;[S515]	-0.71	-0.55
PDLIM1	O00151	1xPhospho;[S130]	-0.65	-0.65
AKT1S1	Q96B36	1xPhospho;[S183]	-0.57	-0.66
CBX5	P45973	1xPhospho;[S92]	-0.60	-0.60
CD44	P16070	1xPhospho;[S706]	0.59	0.71
ATXN2L	Q8WWM7	1xPhospho;[S684]	-0.90	-0.68
ATXN2L	Q8WWM7	1xPhospho;[T/S_678-688]	-0.83	-0.97
ABLIM1	A0A0A0MRL6	1xPhospho;[S623]	0.56	0.55
BAIAP2L1	Q9UHR4	1xPhospho;[S261]	-0.68	-0.60
CXADR	P78310	1xPhospho;[S332]	-0.54	-0.59
MBD3	K7EIE8	1xPhospho;[S29]	-0.53	-0.53
TNKS1BP1	Q9C0C2	2xPhospho;[S691];[S695]	-0.56	-1.40
RPS6	P62753	2xPhospho;[S235];[S240]	-0.66	-0.92
AHNAK	Q09666	1xPhospho;[S135]	-2.40	-0.93
SETX	Q7Z333	1xPhospho;[S1366]	-0.80	-0.69
REPS1	Q96D71	1xPhospho;[S709]	-1.13	-0.75
PLCH1	Q4KWH8	1xPhospho;[S1307]	-0.64	-0.60
PCYT1A	P49585	1xPhospho;[S347]	-0.94	-0.96
RPS6	P62753	3xPhospho;[S235];[S236];[S240]	-1.73	-1.70
RRM2	P31350	1xPhospho;[S20]	-0.93	-1.16
ACIN1	Q9UKV3	1xPhospho;[S240]	-0.73	-0.62
PRRC2C	Q9Y520	1xPhospho;[S2143]	-0.61	-0.55
TP53	A0A087WXZ1	1xPhospho;[S156]	-1.20	-0.81
ITGB4	P16144-2	1xPhospho;[S1364]	-0.72	-0.59
ZNF428	Q96B54	2xPhospho;[S99];[T108]	-0.51	-1.06
MKI67	P46013	1xPhospho;[S1679]	-0.53	-1.14
VAC14	Q08AM6	1xPhospho;[S509]	-0.66	-0.67
MAP4K4	O95819-6	1xPhospho;[S625]	-0.56	-0.72
ICE1	Q9Y2F5	1xPhospho;[S533]	-0.58	-0.62
LARP1	Q6PKG0	1xPhospho;[T376]	-0.90	-0.53
ACACA	Q13085	2xPhospho;[S23];[S29]	-1.29	-0.54
GPATCH8	Q9UKJ3	1xPhospho;[S1107]	-1.28	-0.88
YTHDC2	Q9H6S0	1xPhospho;[S1202]	-0.66	-0.75
RANGAP1	P46060	2xPhospho;[S442];[S/T_414-445]	-0.57	-1.37
TMPO	P42167	2xPhospho;[T154];[S156]	0.59	1.04
RPS6	P62753	2xPhospho;[S240];[S/T_234-243]	-1.34	-0.55
ATXN2L	Q8WWM7	1xPhospho;[S449]	-0.57	-0.60
SHROOM2	Q13796	1xPhospho;[S974]	-0.76	-0.51
IGF2BP1	Q9NZI8	1xPhospho;[S181]	-1.09	-1.32
F2RL1	P55085	1xPhospho;[S373]	-0.74	-0.75
MAP4	E7EVA0	1xPhospho;[S1932]	-0.76	-0.84
RPS6	P62753	2xPhospho;[S235];[S240]	-0.97	-0.75
PPP1R12A	O14974	2xPhospho;[S472];[S473]	-0.54	-0.87

ANKRD17	O75179	2xPhospho;[S1696];[S]	-0.56	-0.67
NFIX	C9JWJ8	1xPhospho;[S268]	-0.70	-0.60
PROSER2	Q86WR7	1xPhospho;[S43]	-0.58	-0.70
ZNF814	B7Z6K7	1xPhospho;[S87]	-0.63	-0.85
DYNC1LI1	Q9Y6G9	1xPhospho;[T408]	-0.58	-0.53
INTS3	Q68E01	1xPhospho;[S995]	-0.92	-1.46
RC3H2	Q9HBD1	2xPhospho;[S803];[S808]	-0.88	-0.92
AHNAK	Q09666	1xPhospho;[S41]	-0.50	-0.91
SUB1	P53999	1xPhospho;[S10]	0.51	0.58
ARHGEF2	V9GYM8	1xPhospho;[S167]	-0.59	-0.75
HBS1L	Q9Y450	1xPhospho;[S67]	-0.71	-1.12
JPT2	Q9H910	1xPhospho;[T76]	-0.63	-0.53
SUGT1	Q9Y2Z0	1xPhospho;[T265]	-0.53	-0.73
CARMIL2	Q6F5E8	1xPhospho;[S1381]	-0.56	-0.68
IWS1	Q96ST2	1xPhospho;[T725]	-1.33	-0.82
MKI67	P46013	1xPhospho;[T1017]	-0.56	-0.76
IGF2BP1	Q9NZI8	1xPhospho;[S181]	-1.07	-1.13
IWS1	Q96ST2	1xPhospho;[S720]	-1.26	-0.60
NAV1	Q8NEY1	1xPhospho;[S452]	-0.76	-0.62
GYS1	P13807	1xPhospho;[S727]	-0.50	-1.08
SPICE1	Q8N0Z3	1xPhospho;[S/T_809-829]	0.53	0.56
TRA2A	Q13595	1xPhospho;[T88]	1.18	0.68
AUTS2	Q75MD7	1xPhospho;[S708]	-1.23	-0.73
SRSF1	J3KTL2	1xPhospho;[S199]	-0.70	-0.89
ARHGEF12	Q9NZN5	1xPhospho;[T/S/Y_1284-1304]	-0.56	-1.13
MAGED2	Q9UNF1	1xPhospho;[T72]	-0.68	-0.55
RPS6	P62753	2xPhospho;[S235];[S236]	-1.08	-1.38
IWS1	Q96ST2	1xPhospho;[S/T_718-727]	-0.58	-0.51
HNRNPD	Q14103	1xPhospho;[S80]	0.80	0.60
SUGT1	Q9Y2Z0	1xPhospho;[T284]	-0.55	-0.56
UTF1	Q5T230	1xPhospho;[S18]	-1.01	-0.54
SRRM2	Q9UQ35	1xPhospho;[S952]	0.79	0.59
TNRC6B	Q9UPQ9	1xPhospho;[S567]	0.53	0.53
USP1	O94782	1xPhospho;[S327]	-0.67	-0.60
KIFC1	Q9BW19	1xPhospho;[S31]	-0.73	-1.02
SSBP3	Q9BWW4	1xPhospho;[S347]	-0.95	-0.60
SRRM2	Q9UQ35	1xPhospho;[S1729]	0.63	0.93
COBLL1	Q53SF7	1xPhospho;[S364]	-0.56	-0.55
PPP4R3A	Q6IN85	1xPhospho;[S741]	-0.62	-0.89
FGD3	Q5JSP0	1xPhospho;[S128]	-1.26	-0.97
ATRX	P46100	3xPhospho;[S1990];[S1991];[S/T/Y_1988-2003]	0.50	0.75
PRR14L	Q5THK1	1xPhospho;[S]	-1.13	-1.12
FAM169A	D6RB01	1xPhospho;[S466]	-0.69	-0.66
MICALL1	Q8N3F8	1xPhospho;[T313]	-0.94	-0.79
RAB11FIP1	Q6WKZ4	1xPhospho;[S]	0.74	0.90
SERBP1	Q8NC51	1xPhospho;[S197]	-0.53	-0.51
KTN1	Q86UP2	1xPhospho;[S]	0.84	0.51
SMARCAD1	Q9H4L7	3xPhospho;[S95];[S96];[S98]	-1.05	-1.11
MICALL1	Q8N3F8	1xPhospho;[S309]	-1.47	-0.93
BZW2	B5MCH7	1xPhospho;[S338]	-0.89	-0.76
GTF3C1	Q12789	2xPhospho;[S1062];[S1068]	-1.01	-0.53
OBI1	Q5W0B1	1xPhospho;[S461]	-0.88	-0.91
IGF2BP1	Q9NZI8	1xPhospho;[T446]	-1.14	-0.63
TOR4A	Q9NXH8	1xPhospho;[S63]	-0.77	-0.69
TMPO	P42167	1xPhospho;[S/T_149-173]	0.71	0.74
IWS1	Q96ST2	1xPhospho;[S720]	-1.03	-0.91
NPY1R	P25929	1xPhospho;[S368]	0.82	1.10
FOXO6	A8MYZ6	1xPhospho;[S210]	-0.86	-0.76
NAV3	Q8IVL0	1xPhospho;[S542]	-0.54	-0.79
ZNF106	Q9H2Y7	1xPhospho;[S861]	-0.58	-0.70
BRSK2	Q8IWQ3-4	1xPhospho;[S367]	-0.64	-0.88
DCLK1	O15075	2xPhospho;[S327];[S332]	-1.21	-0.62
KRT18	P05783	1xPhospho;[S18]	-1.13	-0.61
SRRM2	Q9UQ35	2xPhospho;[S2067];[S2071]	0.70	0.51
SYN3	O14994	1xPhospho;[S484]	-0.59	-0.57
CDC25C	P30307	1xPhospho;[S216]	-0.60	-0.74

CASK	O14936-4	1xPhospho;[S577]	-0.57	-1.29
PXN	F5GZ78	1xPhospho;[S104]	-0.65	-1.09
SRPK2	P78362	2xPhospho;[S494];[S496]	-0.69	-1.21
ZNF711	Q9Y462	1xPhospho;[S459]	-0.50	-1.10
THOC2	Q8NI27	1xPhospho;[S/T_1385-1397]	-0.56	-0.66
SRSF7	Q16629	1xPhospho;[S192]	0.64	0.66
EHBP1	Q8NDI1	1xPhospho;[S1058]	-0.75	-1.28
SERBP1	Q8NC51	1xPhospho;[S234]	-0.70	-1.01
GTSE1	Q9NYZ3	1xPhospho;[S243]	-0.74	-0.76
CTTNBP2	Q8WZ74	1xPhospho;[S]	-0.71	-0.65
SRRM2	Q9UQ35	2xPhospho;[S1072];[S1073]	0.53	0.87
SRRM2	Q9UQ35	1xPhospho;[S1657]	0.54	1.00
SRRM2	Q9UQ35	2xPhospho;[S1657];[S1658]	0.78	0.55
LMNB1	P20700	1xPhospho;[S393]	-0.61	-1.19
AHNAK	Q09666	1xPhospho;[S5870]	1.17	1.07
PJA1	Q8NG27	1xPhospho;[S265]	-0.57	-0.67
SAMSN1	Q9NSI8	1xPhospho;[S34]	1.11	0.52
EFHD2	Q96C19	1xPhospho;[S]	-0.72	-0.71
JPT2	Q9H910	1xPhospho;[S144]	-0.80	-0.83
RSRC2	Q7L4I2	2xPhospho;[T220];[S222]	1.00	0.64
NUP133	Q8WUM0	1xPhospho;[T63]	-1.01	-0.56
ZRANB2	O95218-2	1xPhospho;[S/Y_273-285]	1.21	1.07
WDR62	O43379	1xPhospho;[S49]	-0.63	-0.83
DCAF10	Q5QP82	1xPhospho;[S349]	-0.78	-0.70
CRMP1	Q14194	2xPhospho;[S518];[S521]	-0.99	-0.86
OSBPL6	Q9BZF3	1xPhospho;[S9]	-0.80	-1.77
RPS6	P62753	2xPhospho;[S/T_233-243]	-2.10	-1.09
SRRM2	Q9UQ35	1xPhospho;[S/T_1644-1664]	0.78	0.91
EFHD2	Q96C19	1xPhospho;[S]	-0.51	-0.59
DSP	P15924	2xPhospho;[S165];[S166]	-1.69	-0.69
PFKP	Q01813	1xPhospho;[S386]	-0.61	-0.61
TNS1	A0A087WWW7	1xPhospho;[S756]	-0.60	-1.30
LMO7	Q8WWI1-3	1xPhospho;[S1259]	0.69	0.58
KIF1B	O60333-3	1xPhospho;[S1053]	-0.62	-0.56
TCEA1	A0A1W2PRL9	1xPhospho;[S79]	0.84	2.55
CDCP1	Q9H5V8	1xPhospho;[S797]	-0.75	-0.79
KAT7	O95251	1xPhospho;[S56]	0.74	0.60
CDC42EP1	Q00587	3xPhospho;[S101];[S106];[S113]	-0.59	-1.17
CASKIN1	Q8WXD9	1xPhospho;[S826]	-0.64	-0.58
SRRM2	Q9UQ35	2xPhospho;[S2032];[T2034]	0.74	1.02
ZEB1	P37275	1xPhospho;[T324]	-0.64	-0.80
ABLIM1	A0A0A0MRL6	1xPhospho;[T621]	1.40	0.50
TCOF1	Q13428	1xPhospho;[S771]	0.98	0.71
NECTIN1	Q15223	1xPhospho;[S422]	-0.52	-1.03
GLIS3	Q8NEA6	1xPhospho;[S623]	-0.57	-0.64
INF2	Q27J81	1xPhospho;[T1179]	0.67	0.87
NOM1	Q5C9Z4	1xPhospho;[S280]	-0.51	-0.59
EPB41L1	A0A0C4DH22	2xPhospho;[S546]	-0.72	-1.34
TMPO	P42167	2xPhospho;[S159];[T160]	0.59	0.96
SYN3	O14994	1xPhospho;[S475]	-0.54	-0.63
POF1B	Q8WVV4-1	1xPhospho;[S123]	-0.59	-0.89
CHD4	A0A0C4DGG9	2xPhospho;[S1556];[S1560]	0.60	0.63
SPAG9	O60271	1xPhospho;[T330]	-0.61	-0.51
SATB2	Q9UPW6	1xPhospho;[S587]	0.53	0.55
EEF1D	P29692	1xPhospho;[S162]	-0.73	-1.54
UBR5	O95071	1xPhospho;[S1549]	-0.63	-0.65
PNN	Q9H307	1xPhospho;[S692]	-0.93	-0.84
PDE3A	Q14432	1xPhospho;[T311]	-1.08	-0.61
NHSL1	Q5SYE7-2	1xPhospho;[S]	-0.73	-0.83
SRSF6	Q13247	2xPhospho;[S299];[S303]	0.72	0.64
SRRM2	Q9UQ35	1xPhospho;[S1499]	0.76	0.90
CHERP	Q8IWX8	2xPhospho;[S817];[T819]	0.63	0.85
SRRM2	Q9UQ35	2xPhospho;[S2044];[S2046]	0.90	1.19
SH3KBP1	Q96B97	1xPhospho;[S181]	-0.79	-0.80
UBAP2L	Q14157	3xPhospho;[S475];[S476];[S477]	-0.55	-1.39
SRRM2	Q9UQ35	1xPhospho;[S957]	0.69	0.54

ZC3HC1	Q86WB0	1xPhospho;[T373]	-0.72	-0.85
PTBP1	A0A0U1RRM4	1xPhospho;[S155]	-0.70	-0.81
SALL1	Q9NSC2	1xPhospho;[S586]	-0.67	-1.22
ATG13	O75143	1xPhospho;[S355]	0.78	0.63
PRKACA	K7ERP6	1xPhospho;[T/Y/S_151-169]	0.61	0.74
PLEKHA3	Q9HB20	1xPhospho;[T/S/Y_242-265]	-0.78	-0.61
PRRC2C	Q9Y520	3xPhospho;[T1244];[S1246];[S1248]	-0.53	-0.88
RSRC2	Q7L4I2	2xPhospho;[S222];[S/T216-227]	0.96	1.26

Experiment two (30min and 24h timepoints): NCI-H1975, RKO, MCF-7 (Temsirolimus, NF1 knockout as resistance mechanism), KBM7/HAP1 (both drugs), A549, T47D, and BT474 cell lines.

Gene name	Uniprot	Phosphorylation sites	Log2 FC 30m	Log2 FC 24h
UBE2O	Q9C0C9	1xPhospho;[S839]	-0.75	-0.61
RPS6	P62753	2xPhospho;[S235];[S240]	-0.67	-0.53
RPS6	P62753	3xPhospho;[S235];[S236];[S240]	-0.58	-1.35
PFKFB3	A0A1W2PR17	2xPhospho;[S461];[S467]	-0.57	-0.86
NECAP2	Q9NVZ3	1xPhospho;[S/T_177-195]	0.60	0.55
AKAP12	Q02952	1xPhospho;[S514]	-3.04	-0.93
AKAP13	Q12802	1xPhospho;[S1565]	0.50	0.53
AUP1	Q9Y679	1xPhospho;[S354]	-0.62	-1.27
BICRA	A0A087WWH3	1xPhospho;[S703]	0.63	0.58
BRCA2	P51587	1xPhospho;[S93]	-1.51	-0.99
BCAS3	K7ESE9	1xPhospho;[S251]	0.51	1.01
CTNND1	C9JZR2	2xPhospho;[S349];[S352]	0.54	0.53
NCAPD2	Q15021	1xPhospho;[S1330]	-2.06	-0.80
CUEDC2	Q9H467	1xPhospho;[S110]	-0.67	-0.71
CREB1	P16220	1xPhospho;[S111]	0.85	0.56
DOCK11	Q5JSL3	1xPhospho;[S440]	1.05	0.61
DTL	Q9NZJ0	1xPhospho;[S679]	-0.69	-1.05
VCPIP1	Q96JH7	1xPhospho;[S747]	0.73	0.64
RFX7	Q2KHR2	1xPhospho;[S321]	-0.51	-0.75
CD3EAP	O15446	1xPhospho;[T287]	-0.51	-1.24
DNMBP	A0A1B0GTX1	1xPhospho;[S119]	1.03	0.56
DTX3L	Q8TDB6	1xPhospho;[S532]	0.61	0.76
HECTD1	Q9ULT8	1xPhospho;[S/T_1382-1403]	-1.19	-0.73
EVL	Q9UI08	1xPhospho;[S331]	1.02	0.83
ERRFI1	Q9UJM3	1xPhospho;[S369]	-0.88	-1.33
EIF4G1	E7EX73	1xPhospho;[S541]	0.81	0.51
EIF4G1	Q04637	1xPhospho;[S704]	0.80	0.58
BRIP1	Q9BX63	1xPhospho;[S1237]	-0.88	-1.33
FEZ2	Q9UHY8	1xPhospho;[S29]	-1.44	-0.75
FHOD1	Q9Y613	1xPhospho;[S523]	-0.58	-1.08
ADD3	Q9UEY8	1xPhospho;[S677]	0.57	0.63
IFI16	Q16666	1xPhospho;[S153]	0.59	0.72
GTF2F1	M0QXD6	1xPhospho;[S349]	0.50	0.66
GLCCI1	Q86VQ1	1xPhospho;[S303]	1.08	1.04
GAPDH	P04406	1xPhospho;[S266]	-0.68	-0.69
HBS1L	Q9Y450	1xPhospho;[S127]	-0.70	-0.72
HTATSF1	O43719	1xPhospho;[S481]	-0.89	-0.55
IVNS1ABP	Q9Y6Y0	1xPhospho;[S277]	-0.74	-0.99
PPIP5K2	O43314	1xPhospho;[S1006]	0.53	0.54
KANSL3	Q9P2N6	2xPhospho;[S511];[S515]	1.00	1.02
KRT18	P05783	1xPhospho;[S18]	0.69	0.58
KRT19	P08727	1xPhospho;[S35]	0.72	0.76
KRT8	P05787	1xPhospho;[S475]	-0.67	-0.77
KRT8	P05787	2xPhospho;[S21];[S24]	0.82	0.72
KNL1	Q8NG31	1xPhospho;[S1076]	-0.51	-0.90
KANK2	Q63ZY3	1xPhospho;[S540]	-0.65	-0.71
LARP4	Q71RC2	1xPhospho;[S647]	-0.58	-0.62
LIMA1	Q9UHB6	1xPhospho;[S374]	-0.59	-0.86
LIMA1	Q9UHB6	1xPhospho;[S698]	-0.53	-0.67
KDM3B	Q7LBC6	1xPhospho;[S/T_725-763]	0.60	0.51
MAP7D3	Q8IWC1	1xPhospho;[S524]	-0.64	-1.69
NCKAP5L	Q9HCH0	1xPhospho;[S436]	-0.78	-0.84
AHNAK	Q09666	1xPhospho;[S135]	-0.58	-1.16

FAM129B	Q96TA1	1xPhospho;[S624]	0.67	1.15
NASP	P49321	1xPhospho;[S421]	-0.78	-0.65
NASP	P49321	1xPhospho;[T404]	-0.67	-0.50
NCBP1	Q09161	1xPhospho;[S22]	-1.05	-1.01
NFATC2	Q13469	2xPhospho;[S236];[S243]	0.72	0.65
NUP153	P49790	1xPhospho;[T515]	-0.65	-0.55
NUP50	Q9UKX7	1xPhospho;[S221]	-1.10	-0.77
NOLC1	A0A0A0MRM9	2xPhospho;[T616];[T619]	-0.85	-1.30
NCL	P19338	1xPhospho;[T121]	-0.52	-1.33
OSTF1	Q92882	1xPhospho;[S213]	0.88	0.90
PALLD	Q8WX93	1xPhospho;[S893]	-1.05	-1.07
SERBP1	Q8NC51	1xPhospho;[S234]	-0.65	-0.71
PLEKHA3	Q9HB20	1xPhospho;[S289]	-0.71	-0.83
PBX2	P40425	1xPhospho;[S330]	0.56	0.77
AKT1S1	Q96B36	1xPhospho;[S183]	-0.79	-0.73
PPP1R12A	O14974	2xPhospho;[S862];[S871]	0.56	0.57
SUGT1	Q9Y2Z0	1xPhospho;[T265]	-0.63	-0.59
SPDL1	Q96EA4	1xPhospho;[S555]	-0.74	-1.21
RABL6	Q3YEC7	2xPhospho;[S470];[S471]	0.90	0.60
RAF1	P04049	2xPhospho;[S301];[S/T_283-309]	-0.96	-0.60
RTN3	O95197	1xPhospho;[S246]	-0.64	-1.02
RPS6KB1	P23443	2xPhospho;[T444];[S447]	-1.02	-0.92
RPS6KB1	P23443	3xPhospho;[S441];[T444];[S447]	-0.53	-0.85
RNF219	Q5W0B1	2xPhospho;[S525];[S526]	-0.74	-1.57
RRN3	Q9NYV6	1xPhospho;[S44]	-0.54	-0.76
PPP6R1	Q9UPN7	1xPhospho;[S877]	-0.58	-0.53
SIPA1L1	O43166	2xPhospho;[S1564];[S1568]	-0.70	-0.54
SSFA2	P28290	2xPhospho;[T87];[S92]	1.05	1.29
SKA2	J3KTC5	1xPhospho;[S72]	-0.59	-0.52
SKA3	Q8IX90	1xPhospho;[S155]	-0.78	-1.35
SPRED2	C9JG63	1xPhospho;[S221]	-1.22	-0.63
TTC7A	Q9ULT0	1xPhospho;[S51]	0.60	0.57
TCOF1	Q13428	1xPhospho;[S1410]	-1.25	-0.54
TCOF1	Q13428	1xPhospho;[T310]	-0.99	-1.32
LYN	P07948	1xPhospho;[S13]	1.36	0.67
SORBS3	O60504	1xPhospho;[S530]	0.71	0.52
YAF2	Q8IY57	1xPhospho;[S163]	1.07	0.52
ZNHIT2	Q9UHR6	1xPhospho;[T161]	-0.56	-0.70
ZNF740	Q8NDX6	1xPhospho;[S44]	0.54	0.60

Tablle S4. Custom oligos used for generating mutant cell lines.

Codon for PIK3CA H1047 is underlined in the HDR template. Two additional silent mutations (lower case) were introduced in the protospacer sequence to prevent further cutting by Cas9

Name	Sequence 5'-3'
**HDR templates**	
PIK3CA H1047R	AGACCCTAGCCTTAGATAAAACTGAGCAAGAGGCTTTGGAGTATTTCATGAAACAAATGAATGATGCcCgTCAcGGTGGCTGGACAACAAAAATGGATTGGATCTTCCACACAATTAAACAGCATGCATTGAACTGAAAAG
**crRNA sequences**	
PTEN_knockout_1	AGAGCGTGCAGATAATGACA
PTEN_knockout_2	CCAATTCAGGACCCACACGA
NF1_knockout	GGTCCAGTCAGTGAACGTAA
RB1_knockout	TGAACTACTTACGAACTGCT
PIK3CA_H1047R	ATGAATGATGCACATCATGG

Table S5. Custom oligos used for CGE assay.
HDR oligos were ordered with equimolar ratios for randomized nucleotides (underlined), unless indicated otherwise in brackets, according to the specifications for hand-mixed bases from Integrated DNA Technologies.
For RPS6 and NOLC1 double phosphorylation site mutations, as well as NOLC1 and TCOF1 E-box mutations, the HDR templates (WT and mutated) are transfected simultaneously as an equimolar mixture. For all the other phosphorylation site mutations, randomized template generates WT, non-phosphorylatable, and phosphomimetic edits.

**Name**	**Sequence 5'-->3'**
**HDR templates**	
HK2_T473	TGACAGCAGTGGCTTACCGGCTGGCCGATCAACACCGTGC**N**CG**N**CA**R**AA**RRM**A**Y**T**R**GAGCATCTGCAGCTGAGCCATGACCAGCTGCTGGAGGTCA
RPS6_S235_S236_WT	AGGCTAAGGAGAAGCGCCAGGAACAAATTGCGAAGAGACG**N**AG**R**CT**N**TCCTCTCT**N**CG**N**GCTTCTACTTCTAAGTCTGAATCCAGTCAGAAATAAGATT
RPS6_S235_S236_Mutated_AA	AGGCTAAGGAGAAGCGCCAGGAACAAATTGCGAAGAGACG**N**AG**R**CT**N**GCCGCTCT**N**CG**N**GCTTCTACTTCTAAGTCTGAATCCAGTCAGAAATAAGATT
RPS6_S235_S236_Mutated_DD	AGGCTAAGGAGAAGCGCCAGGAACAAATTGCGAAGAGACG**N**AG**R**CT**N**GACGATCT**N**CG**N**GCTTCTACTTCTAAGTCTGAATCCAGTCAGAAATAAGATT
EIF4EBP1_T37	TGCTCGGCGACGGCGTGCAGCTCCCGCCCGGGGACTACAG**Y**AC**NRM**CCC**N**GG**N**GG**N**ACGCTCTTCAGCACCACCCCGGGAGGTAGGCGCGGGCTTG
AKT1S1_S183	CAGTGCCCCCAGCCTCAGCCCTACCCACACAGCAGTACGCCAA**RKMY**CT**N**CC**N**GT**N**TCTGTGCCCGTCTGGGGCTTCAAGGAGAAGAGGACAGAGG
EIF4G1_S704	TCTTGTCTTCATCCCTTGCTTAGCAGGCTGGCCTGGGACC**N**CG**N**CG**NKM**TCA**R**CA**R**GGACCCCGAAAAGAACCACGCAAGATCATTGCCACAGTGT
RRN3_S44	ACAGGATTTCAAATATGCGTGCATTAGAGAATGACTTTTT**Y**AA**YKM**TCC**N**CC**NM**G**R**AAAACTGTTCGGTTTGGTGGAACTGTGACAGAAGTCTTGC
TCOF1_S1410	AAGCAAGTGGTGATGTCAAGGAGAAGAAAGGGAAGGGGTC**N**CT**N**GG**NKM**CCA**R**GG**N**GCCAAGGACGAGCCAGAAGAGGAGCTTCAGAAGGGGATGG
NOLC1_T607_T610_WT	CCAAGGAGGCAGAGACTCCTCAGGCCAAGAAGATAAAGCT**N**CA**R**AC**Y**CC**N**AA**Y**ACATT**Y**CCAAAAAGGAAGAAAGTAAGTTGTCTCACTTTCTTCTCAG
NOLC1_T607_T610_Mutated_AA	CCAAGGAGGCAGAGACTCCTCAGGCCAAGAAGATAAAGCT**N**CA**R**GC**Y**CC**N**AA**Y**GCATT**Y**CCAAAAAGGAAGAAAGTAAGTTGTCTCACTTTCTTCTCAG
NOLC1_T607_T610_Mutated_DD	CCAAGGAGGCAGAGACTCCTCAGGCCAAGAAGATAAAGCT**N**CA**R**GA**Y**CC**N**AA**Y**GACTT**Y**CCAAAAAGGAAGAAAGTAAGTTGTCTCACTTTCTTCTCAG
RPA34_S285_T287	AGGAACAGATTAACACTGAGCCTCTAGAAGACACAGTCCT**NKMY**CC**NRMN**AA**R**AA**R**AGAAAGAGGCAAAAGGGGACGGAAGGGATGGAGCCAGAGG
NOLC1_E-box_WT	TGAGTCTTGTTGCTTTTTTCTTGTGACCTGTGCAGCGGC**(N3:08087608) (N3) (N4:08760808)**AGCGTG**(N3) (N3) (N1:76080808) (N3) (N4)**CTCGTG**(N3) (N4)**CCCAAGCCACATGGCTGGCAGAGTGCAGCGGGGAGGTAG
NOLC1_E-box_Mutated	TGAGTCTTGTTGCTTTTTTCTTGTGACCTGTGCAGCGGC**(N3:08087608) (N3) (N4:08760808)**TATTTA**(N3) (N3) (N1:76080808) (N3) (N4)**TATTTA**(N3) (N4)**CCCAAGCCACATGGCTGGCAGAGTGCAGCGGGGAGGTAG
TCOF1_E-box_WT	CACGCCCTTCCTGGTAGCGGGTATTTTAAGTTTCCTAAGTCT**(N4:08760808) (N4) (N4) (N3:08087608) (N4)**CACGTG**(N3) (N4) (N2:08080876) (N1:76080808) (N3)**GCTCTGCGCGGCCCCCCCTGGGGCAAGGAGGTTGCTGCGAGT
TCOF1_E-box_Mutated	CACGCCCTTCCTGGTAGCGGGTATTTTAAGTTTCCTAAGTCT**(N4:08760808) (N4) (N4) (N3:08087608) (N4)**TATTTA**(N3) (N4) (N2:08080876) (N1:76080808) (N3)**GCTCTGCGCGGCCCCCCCTGGGGCAAGGAGGTTGCTGCGAGT

crRNA sequences

HK2_T473	GCGCATCTCCTCCATGTAGC
RPS6_S235_S236	AGAAGTAGAAGCTCGCAGAG
EIF4EBP1_T37	GACTACAGCACGACCCCCGG
AKT1S1_S183	GACGGGCACAGACACAGGCA
EIF4G1_S704	GGTCCCTGCTGAGAGCGCCG
RRN3_S44	AACCGAACAGTTTTTCTTGG
TCOF1_S1410	AGGGGTCTCTTGGCTCCCAA
NOLC1_T607_T610	CCTTTTTGGAAATGTGTTAG
RPA34_S285_T287	CCTCTTTCTCTTTTTGGTCG
NOLC1_E-box	CCATGTGGCTTGGGGCCACG
TCOF1_E-box	AAGTCTCCCGCCACGTGGCT

pegRNAs

CDK1_Y15	ACCCTTATACACAACTCCATGTTTTAGAGCTAGAAATAGCAAGTTAAAATAAGGCTAGTCCGTTATCAACTTGAAAAAGTGGCACCGAGTCGGTGCTTGTTTCAGG**N**AC**NKWY**GGAGTTGTGTATAA
HK2_Y461	TGGTGACAGCAGTGGCTTACGTTTTAGAGCTAGAAATAGCAAGTTAAAATAAGGCTAGTCCGTTATCAACTTGAAAAAGTGGCACCGAGTCGGTGCGGTGTTGATCGGC**N**AG**N**CGG**KM**AGCCACTGCTGTC
SRSF1	GATCGAGATCTTCCATAACTGTTTTAGAGCTAGAAATAGCAAGTTAAAATAAGGCTAGTCCGTTATCAACTTGAAAAAGTGGCACCGAGTCGGTGCATGGGCCCAG**RRVY**CC**N**AG**Y**TATGGAAGATCTC

Primers for gDNA amplification. Target-specific sequence underlined

CDK1_Y15_FP	ACACGACGCTCTTCCGATCTAGATCTTTAGTTTGTGGGGTGTG
CDK1_Y15_RP	GACGTGTGCTCTTCCGATCTGGATGACGAAGTTCCTTTAATAGAG
HK2_Y461_T473_FP	ACACGACGCTCTTCCGATCTGCAGTGGCAAAGGTGCAG
HK2_Y461_T473_RP	GACGTGTGCTCTTCCGATCTGGCACTGGCATGAGTCTCC
RPS6_S235_S236_FP	ACACGACGCTCTTCCGATCTGGCCTTAACTGTTTGCTG
RPS6_S235_S236_RP	GACGTGTGCTCTTCCGATCTGACCTAACTTTCCCTCTCTTC
EIF4EBP1_T37_FP	ACACGACGCTCTTCCGATCTTGCAGCGCACAGGAGAC
EIF4EBP1_T37_RP	GACGTGTGCTCTTCCGATCTCCGGTCCAATCCGCGATTC
AKT1S1_S183_FP	ACACGACGCTCTTCCGATCTAGTCCACCCTCTCTTTCAGATG
AKT1S1_S183_RP	GACGTGTGCTCTTCCGATCTTCACCGGCCCATTCTCCTC
EIF4G1_S704_FP	ACACGACGCTCTTCCGATCTCGTGTAGTAGTGGTGTCACATATTG
EIF4G1_S704_RP	GACGTGTGCTCTTCCGATCTCTGGGTTTCCAGGCTTTCTC
SRSF1_S199_FP	ACACGACGCTCTTCCGATCTCTATCCAATAGGGAGAAACTGC
SRSF1_S199_RP	GACGTGTGCTCTTCCGATCTAATAGCGTGGTGATCCTCTG
RRN3_S44_FP	ACACGACGCTCTTCCGATCTAGCTTGTTAATCTTGGTTCCAC
RRN3_S44_RP	GACGTGTGCTCTTCCGATCTGCAATAGTTGTATTCTGACCTAACC
TCOF1_S1410_RP	ACACGACGCTCTTCCGATCTGGGAAAGCAAAGAGAGACAAAG
TCOF1_S1410_FP	GACGTGTGCTCTTCCGATCTGATCTCCACCTTCAACCGTC
NOLC1_T607_T610_FP	ACACGACGCTCTTCCGATCTAGAAGCGGAAGCAGAATGAGG
NOLC1_T607_T610_RP	GACGTGTGCTCTTCCGATCTGCAGAGCTGTCAAGATGATCCTAG
RPA34_S285_T287_FP	ACACGACGCTCTTCCGATCTGAGCCAGAAGACAAGACAGTGAAG
RPA34_S285_T287_RP	GACGTGTGCTCTTCCGATCTCTCCATCATTGCCATCTGTCC
NOLC1_E-box_FP	ACACGACGCTCTTCCGATCTAGTGACCAGAGAAAGCCTGC
NOLC1_E-box_RP	GACGTGTGCTCTTCCGATCTCCGAAGAACCAACGTCCTCA
TCOF1_E-box_FP	ACACGACGCTCTTCCGATCTCAGGGGCCGAACTTTGTAAT
TCOF1_E-box_RP	GACGTGTGCTCTTCCGATCTCTAAGAGGCTGTAGGGTCCC

Table S6. Gene regulatory Network query results.
Top 100 results for each query are listed

Query 1: Results for ChIP-seq query of paralog corresponding to **Fig. 1C** left panel

Target	Band	Score	p-value
ROCK1;LATS1;LATS2;ROCK2	18:q11.1;6:q25.1;13:q12.11;2:p25.1	2.2E-19	0.00038
KIAA1772;ENSG00000196208	18:q11.1;2:p25.1	5.34E-19	0.0008
MYC;MYCL2;MYCL1;MYCN	8:q24.21;X:q22.3;1:p34.2;2:p24.3	5.81E-19	0.00087
CXADR;C1orf204;ENSG00000166250	21:q21.1;1:q23.2;11:q24.1	9.14E-19	0.00139
ENSG00000216205	18:q11.1	1.92E-18	0.00266
AKAP13;ARHGEF18;ARHGEF2;ENSG00000214944;ENSG00000038102	15:q25.3;19:p13.2;1:q22;5:q13.2;5:q13.2	2.22E-18	0.00309
IRS2;IRS4;IRS1	13:q34;X:q22.3;2:q36.3	1.93E-17	0.01989
ZNF536;ZNF217;ZNF516;ZNF219	19:q12;20:q13.2;18:q23;14:q11.2	2.11E-17	0.02133
ENSG00000210669	8:p12	3.01E-17	0.02873
TPH2;TH;PAH;TPH1	12:q21.1;11:p15.5;12:q23.2;11:p15.1	3.28E-17	0.03102
SEMA3G;SEMA3B;SEMA3D;SEMA3A;SEMA3E	3:p21.1;3:p21.31;7:q21.11;7:q21.11;7:q21.11	3.42E-17	0.03204
ENSG00000216487	21:q21.1	4.21E-17	0.03801
ANKRD13B;ANKRD13C;ANKRD13A;ANKRD13D	17:q11.2;1:p31.1;12:q24.11;11:q13.1	5.5E-17	0.04732
GOLGA2;GOLGA9P;GOLGA8E;GOLGA8G;GOLGA8F;GOLGA8B;GOLGA8A;GOLGA6B;GOLGA6D;GOLGA6C;ENSG00000205333;ENSG00000187812;ENSG00000215690;ENSG00000181984;ENSG00000185182;ENSG00000215186;ENSG00000186399;ENSG00000188626;ENSG00000206127;ENSG00000178115;ENSG00000215749;ENSG00000213974;ENSG00000197092;ENSG00000197978;ENSG00000196648;ENSG00000188388;ENSG00000103832;ENSG00000205281;ENSG00000184206;ENSG00000186322;ENSG00000156363;ENSG00000214394;ENSG00000214433;ENSG00000179938;ENSG00000188532;ENSG00000196102;ENSG00000167141;ENSG00000166104;ENSG00000215623;ENSG00000197414;ENSG00000215405;ENSG00000220356;ENSG00000174450;ENSG00000205315	9:q34.11;15:q11.2;15:q11.2;15:q13.1;15:q13.1;15:q14;15:q14;15:q24.1;15:q24.2;15:q24.1;15:q24.2;15:q24.2;NT_113925:;15:q11.2;15:q11.2;10:p11.23;NT_113924:;15:q13.1;15:q13.3;15:q13.3;NT_113927:;19:p12;NT_113927:;15:q25.2;15:q25.2;15:q25.3;15:q13.2;15:q25.2;15:q25.2;15:q25.2;15:q25.2;15:q26.3;15:q26.1;15:q13.2;15:q13.3;15:q13.2;15:q24.2;15:q22.2;NT_113924:;15:q11.2;15:q11.2;15:q22.2;15:q11.2;15:q24.3	6.22E-17	0.05245
POTEA;ANKRD36;ANKRD20A5;ANKRD19;ACTBL3;POTEF;POTEE;POTED;POTEG;POTEC;POTEH;ENSG00000196834;ENSG00000222038;ENSG00000222036;ENSG00000155319;ENSG00000181626;ENSG00000187984;ENSG00000213114	8:p11.1;2:q11.2;18:p11.21;9:q22.31;2:q21.1;2:q21.1;2:q21.1;21:q11.2;14:q11.1;15:q11.2;22:q11.1;14:q11.1;2:q21.1;2:q21.1;16:q11.2;18:p11.21;2:q11.1;3:q29	1.04E-16	0.07881
ENSG00000220521	21:q21.1	1.07E-16	0.081
HAS2;HAS1;HAS3	8:q24.13;19:q13.33;16:q22.1	1.09E-16	0.08172
ENSG00000222786	8:q24.13	1.1E-16	0.08254
ENSG00000221315	8:q24.21	1.13E-16	0.08395
M6PRBP1;PLIN;ADFP;ENSG00000214456	19:p13.3;15:q26.1;9:p22.1;19:p13.3	1.22E-16	0.0891
KRT8P9;KRT79;KRT7	12:q13.13;12:q13.13;12:q13.13	1.62E-16	0.11112
ENSG00000213993;ENSG00000215477;ENSG00000214855;ENSG00000213358;ENSG00000213952;ENSG00000214791;ENSG00000214871;ENSG00000215039;ENSG00000215246;ENSG00000215837;ENSG00000214116;ENSG00000214343;ENSG00000214000;ENSG00000214797;ENSG00000214467	19:p13.11;13:q14.12;14:q22.2;2:p11.2;2:q32.1;15:q15.3;7:p15.3;12:p13.31;5:p15.33;3:q29;3:q29;7:q21.2;19:p13.11;11:q12.1;12:q13.13	1.99E-16	0.13003
BTG4;BTG1;BTG2;BTG3	11:q23.1;12:q21.33;1:q32.1;21:q21.1	2.14E-16	0.13698
POU3F1;POU3F4;POU3F3;POU3F2;POU5F1P1;ENSG00000204531	1:p34.3;X:q21.1;2:q12.1;6:q16.2;8:q24.21;6:p21.33	2.35E-16	0.14737
SEMA3C;SEMA3F	7:q21.11;3:p21.31	2.76E-16	0.16579
ENSG00000214789	8:q24.21	2.76E-16	0.1658
TRPM2;TRPM4;TRPM5;TRPM8	21:q22.3;19:q13.33;11:p15.5;2:q37.1	3.03E-16	0.17732

SNORD1C;ENSG00000214865;ENSG00000204758;ENSG00000203831;ENSG00000215107;ENSG00000205039;ENSG00000183154;ENSG00000213990	17:q25.1;4:q23;5:q35.2;10:q25.3;X:q13.2;19:q13.2;8:p12;7:q36.3	3.19E-16	0.18401
C9orf30;TGFA;BTC;TMEFF2	9:q31.1;2:p13.3;4:q13.3;2:q32.3	3.47E-16	0.19537
ENSG00000149617	20:q13.2	3.48E-16	0.19597
ZNF503;ZNF703	10:q22.2;8:p12	3.57E-16	0.19941
KRT73;KRT80;KRT78;KRT72;KRT74;KRT71	12:q13.13;12:q13.13;12:q13.13;12:q13.13;12:q13.13;12:q13.13	3.76E-16	0.20724
ENSG00000212988	8:q24.21	4.63E-16	0.23997
FAM3B;FAM3D;FAM3A;FAM3C	21:q22.3;3:p14.2;X:q28;7:q31.31	4.91E-16	0.24971
THBS1;THBS2	15:q14;6:q27	5.52E-16	0.27026
ARL4A;ARL11;ARL14;ARL4D;ARL4C;ARL4P	7:p21.3;13:q14.3;3:q26.1;17:q21.31;2:q37.1;10:q21.2	5.64E-16	0.27413
ENSG00000209715	18:q11.1	6.16E-16	0.29051
ENSG00000211264	17:q24.3	6.22E-16	0.29236
THRB;NR1I3;NR1I2;VDR;THRA	3:p24.2;1:q23.3;3:q13.33;12:q13.11;17:q21.1	6.42E-16	0.29835
CDK7;CDK4;CDK6;CCRK	5:q13.2;12:q14.1;7:q21.2;9:q22.1	7.11E-16	0.31924
TNS1;TNS4;TENC1;TNS3	2:q35;17:q21.2;12:q13.13;7:p12.3	7.81E-16	0.33841
PDE4DIP;CDK5RAP2;ENSG00000215861	1:q21.1;9:q33.2;1:q21.1	8.04E-16	0.34447
ENSG00000209722	21:q21.1	8.2E-16	0.34918
GPR68;GPR132;GPR65;GPR4	14:q32.12;14:q32.33;14:q31.3;19:q13.32	9.08E-16	0.37194
PNMA5;PNMAL1;ZCCHC12;ZCCHC18;MOAP1;PNMA6B;PNMA6A;PNMA1;PNMA2;PNMA3;ENSG00000204851	X:q28;19:q13.32;X:q24;X:q22.2;14:q32.12;X:q28;X:q28;14:q24.3;8:p21.2;X:q28;19:q13.32	9.33E-16	0.37809
ENSG00000201782	8:q24.21	9.56E-16	0.3841
BCL2L2;BAX;BCL2L7P1;BCL2;BCL2L1	14:q11.2;19:q13.33;6:p21.31;18:q21.33;20:q11.21	9.76E-16	0.38886
PI15;PI16;CRISPLD2;CRISPLD1;R3HDML	8:q21.11;6:p21.2;16:q24.1;8:q21.11;20:q13.12	1.1E-15	0.41617
DDX27;DDX52;DDX49;DDX47	20:q13.13;17:q12;19:p13.11;12:p13.1	1.5E-15	0.49657
HES2;HES6;HES3;HES4;HES1	1:p36.31;2:q37.3;1:p36.31;1:p36.33;3:q29	1.72E-15	0.53272
SH3RF1;SH3RF2;ENSG00000172985	4:q33;5:q32;2:q13	1.73E-15	0.53385
CCND2;CCND1;CCND3	12:p13.32;11:q13.2;6:p21.1	1.74E-15	0.53553
ENSG00000214146	3:q29	1.77E-15	0.53982
BMPER;ENSG00000197558;ENSG00000214028	7:p14.3;7:q36.1;7:q36.1	1.84E-15	0.55032
ENSG00000223215	8:p12	1.86E-15	0.5527
GSDMA;GSDMD;GSDMC	17:q12;8:q24.3;8:q24.21	1.9E-15	0.55984
MIRN1208	8:q24.21	2.12E-15	0.58959
NTSR1;GPR39;NTSR2	20:q13.33;2:q21.2;2:p25.1	2.15E-15	0.59363
FAM65B;FAM65C;FAM65A	6:p22.2;20:q13.13;16:q22.1	2.17E-15	0.59535
RNF215;RNF43;ZNRF3	22:q12.2;17:q22;22:q12.1	2.33E-15	0.61501
PGLYRP3;PGLYRP2;PGLYRP1;PGLYRP4	1:q21.3;19:p13.12;19:q13.32;1:q21.3	2.37E-15	0.61949
ZC3HAV1;TIPARP;PARP11;PARP12	7:q34;3:q25.31;12:p13.32;7:q34	2.45E-15	0.62905
KBTBD11;KLHDC5;KLHDC7A;KLHDC7B	8:p23.3;12:p11.22;1:p36.13;22:q13.33	2.58E-15	0.64237
CD36;SCARB2;SCARB1	7:q21.11;4:q21.1;12:q24.31	2.58E-15	0.64314
RERGL;RERG;RASL11B;RASL11A	12:p12.3;12:p12.3;4:q12;13:q12.2	2.6E-15	0.64421
ENSG00000222110	13:q33.3	2.64E-15	0.64885
KLF9;SP3;KLF14;KLF16;KLF13	9:q21.11;2:q31.1;7:q32.2;19:p13.3;15:q13.3	2.69E-15	0.65385
MIRN29B1	7:q32.3	2.85E-15	0.67004
ENSG00000200783	17:q24.3	2.94E-15	0.67892
TNFRSF19;EDAR;EDA2R	13:q12.12;2:q13;X:q12	3.35E-15	0.71277

RAI14;ANKRD35;ANKRD24;UACA	5:p13.2;1:q21.1;19:p13.3;15:q23	3.41E-15	0.71736
ADRB2;ADRB3;ADRB1	5:q33.1;8:p12;10:q25.3	3.74E-15	0.74213
ACTN2;ACTN4;ACTN3;ACTN1	1:q43;19:q13.2;11:q13.1;14:q24.1	3.91E-15	0.7532
ENSG00000181225	21:q21.1	3.96E-15	0.75649
C9orf3;LTA4H;RNPEPL1;RNPEP	9:q22.32;12:q23.1;2:q37.3;1:q32.1	4.01E-15	0.7593
GRHL3;GRHL1;GRHL2	1:p36.11;2:p25.1;8:q22.3	4.13E-15	0.76709
MTMR4;MTMR3	17:q22;22:q12.2	4.18E-15	0.77047
CAPZA3;CAPZA2;CAPZA1	12:p12.3;7:q31.2;1:p13.2	4.45E-15	0.78578
JPH2;JPH4;JPH3;JPH1	20:q13.12;14:q11.2;16:q24.2;8:q21.11	4.47E-15	0.78699
DUSP22;DUSP9;DUSP7;DUSP6;C20orf57	6:p25.3;X:q28;3:p21.1;12:q21.33;20:q11.21	4.77E-15	0.80241
DLG1;DLG2;DLG4	3:q29;11:q14.1;17:p13.1	4.96E-15	0.81163
NDUFS7;ENSG00000202582;ENSG00000197735;ENSG00000214571;ENSG00000214094;ENSG00000214084;ENSG00000215435;ENSG00000215248;ENSG00000204283;ENSG00000205785;ENSG00000205783;ENSG00000215393;ENSG00000214069	19:p13.3;12:q13.13;10:q26.3;19:p13.3;17:q25.3;17:q25.3;20:q13.33;4:p15.1;17:q25.3;5:p13.1;5:p13.1;15:q11.2;17:q25.3	5.2E-15	0.82195
GABPA;ELK1;ETS2;ETS1	21:q21.3;X:p11.23;21:q22.2;11:q24.3	5.42E-15	0.83087
RHOBTB3;RHOBTB1;RHOBTB2	5:q15;10:q21.2;8:p21.3	5.66E-15	0.84031
RPL21P7;RPL21P69;ENSG00000186244	14:q23.3;13:q12.2;4:q12	5.75E-15	0.84314
RUNX3;RUNX1;RUNX2	1:p36.11;21:q22.12;6:p12.3	5.75E-15	0.84324
CLIC4;CLIC6;CLIC5	1:p36.11;21:q22.12;6:p12.3	5.8E-15	0.84497
TNXA;TNN;TNC;ENSG00000198493	6:p21.32;1:q25.1;9:q33.1;6:p21.32	6.09E-15	0.85493
KLHL30;ENC1;KLHL23;KLHL25	2:q37.3;5:q13.3;2:q31.1;15:q25.3	6.51E-15	0.86793
ZNF79;ZNF391;ZNF286A;ENSG00000204756	9:q33.3;6:p22.1;17:p12;8:q24.3	6.65E-15	0.87185
ENSG00000200754	21:q21.1	7.07E-15	0.88332
PREX1;PREX2	20:q13.13;8:q13.2	7.1E-15	0.88435
ENSG00000211547	2:q21.2	7.27E-15	0.88899
RPS27L;RPS27P9	15:q22.2;1:q21.3	7.33E-15	0.89026
TMEM75	8:q24.21	7.39E-15	0.89178
MYO3B;MYO16;MYO3A	2:q31.1;13:q33.3;10:p12.1	7.93E-15	0.90414
GDAP1;GDAP1L1	8:q21.11;20:q13.12	8.09E-15	0.90761
ENSG00000220883	10:q11.1	8.19E-15	0.9096
SIM1;NPAS1;NPAS3;SIM2	6:q16.3;19:q13.32;14:q13.1;21:q22.13	8.37E-15	0.91289
RFC4;RFC3;RFC2;RFC5	3:q27.3;13:q13.2;7:q11.23;12:q24.23	8.39E-15	0.91347

Query 2: Results for the ChIP-seq query of paralog groups of known cell cycle regulators corresponding to Fig. 1C right panel

**Target**	**Band**	**Score**	**p-value**
MYC;MYCL2;MYCL1;MYCN	8:q24.21;X:q22.3;1:p34.2;2:p24.3	5.81E-19	0.00005
ROCK1;LATS1;LATS2;ROCK2	18:q11.1;6:q25.1;13:q12.11;2:p25.1	1.2E-16	0.00489
CDK7;CDK4;CDK6;CCRK	5:q13.2;12:q14.1;7:q21.2;9:q22.1	7.11E-16	0.02131
CLIC4;CLIC6;CLIC5	1:p36.11;21:q22.12;6:p12.3	5.8E-15	0.10538
C9orf3;LTA4H;RNPEPL1;RNPEP	9:q22.32;12:q23.1;2:q37.3;1:q32.1	1.12E-14	0.16541
GABPA;ELK1;ETS2;ETS1	21:q21.3;X:p11.23;21:q22.2;11:q24.3	1.48E-14	0.19828
TNFRSF19;EDAR;EDA2R	13:q12.12;2:q13;X:q12	1.68E-14	0.21536
BTG4;BTG1;BTG2;BTG3	11:q23.1;12:q21.33;1:q32.1;21:q21.1	2.04E-14	0.24516
PREX1;PREX2	20:q13.13;8:q13.2	2.22E-14	0.25789
THRB;NR1I3;NR1I2;VDR;THRA	3:p24.2;1:q23.3;3:q13.33;12:q13.11;17:q21.1	3.67E-14	0.34677

SAMD8;SGMS2;SGMS1	10:q22.2;4:q25;10:q11.23	3.77E-14	0.35227
BCAS3	17:q23.2	3.96E-14	0.36151
LIMS2;LDB3;LIMS1	2:q14.3;10:q23.2;2:q12.3	5.11E-14	0.415
HIF1A;HIF3A;EPAS1	14:q23.2;19:q13.32;2:p21	1.64E-13	0.69559
CNIH2;CNIH4;CNIH;CNIH3	11:q13.1;1:q42.11;14:q22.2;1:q42.12	2.1E-13	0.7546
ACVRL1;ACVR1	12:q13.13;2:q24.1	2.63E-13	0.80441
TCF7L2;TCF7;LEF1;TCF7L1	10:q25.2;5:q31.1;4:q25;2:p11.2	3.61E-13	0.86716
FAM3B;FAM3D;FAM3A;FAM3C	21:q22.3;3:p14.2;X:q28;7:q31.31	3.71E-13	0.87217
BCKDHB;PDHB	6:q14.1;3:p14.3	4.09E-13	0.88805
ATP13A4;ATP13A3;ATP13A5	3:q29;3:q29;3:q29	4.4E-13	0.89892
RPLP1;RPLP2;RPLP1P4	15:q23;11:p15.5;2:q31.1	6.4E-13	0.94655
KIF20A;KIF23;KIF20B	5:q31.2;15:q23;10:q23.31	6.62E-13	0.94974
MYBL1;MYBL2;MYB	8:q13.1;20:q13.12;6:q23.3	6.92E-13	0.95389
SOX4;SOX6;SOX13;SOX5;SOX12;SOX11	6:p22.3;11:p15.1;1:q32.1;12:p12.1;20:p13;2:p25.2	6.99E-13	0.95485
GLI3;GLI2	7:p14.1;2:q14.2	7.22E-13	0.95792
GDAP1;GDAP1L1	8:q21.11;20:q13.12	7.72E-13	0.96372
SNRPA;SNRPB2	19:q13.2;20:p12.1	8.2E-13	0.96812
PPARG;PPARA;PPARD	3:p25.2;22:q13.31;6:p21.31	8.66E-13	0.9718
COPS5;PSMD14	8:q13.2;2:q24.2	9.76E-13	0.97839
RPL17P33;RPL17P34;ENSG00000215472	15:q23;1:p13.3;18:q21.1	1.08E-12	0.98314
SORBS1;SH3D19;SORBS2;SORBS3	10:q23.33;4:q31.3;4:q35.1;8:p21.3	1.13E-12	0.9849
DOPEY2;DOPEY1	21:q22.12;6:q14.1	1.13E-12	0.98504
KCTD10;KCTD13;TNFAIP1	12:q24.11;16:p11.2;17:q11.2	1.38E-12	0.99178
COPG2;COPG;ENSG00000158623	7:q32.2;3:q21.3;7:q32.2	1.39E-12	0.99178
PLK2;PLK1;PLK3	5:q11.2;16:p12.1;1:p34.1	1.44E-12	0.99276
KIAA1772;ENSG00000196208	18:q11.1;2:p25.1	1.91E-12	0.99721
PRKAG1;PRKAG3;PRKAG2	12:q13.12;2:q35;7:q36.1	2.27E-12	0.99872
RPL21P7;RPL21P69;ENSG00000186244	14:q23.3;13:q12.2;4:q12	2.47E-12	0.99907
RPL36P14	9:q31.3	2.55E-12	0.99913
TEF;DBP;HLF	22:q13.2;19:q13.33;17:q22	2.58E-12	0.9992
EPHB2;EPHB3;EPHB1	1:p36.12;3:q27.1;3:q22.2	2.7E-12	0.99935
PDGFRA;PDGFRB	4:q12;5:q33.1	2.79E-12	0.99943
SEPT4;SEPT1;SEPT5	17:q22;16:p11.2;22:q11.21	2.87E-12	0.99952
UBE3C;KIAA0317;UBE3B	7:q36.3;14:q24.3;12:q24.11	3.13E-12	0.99969
LECT2	5:q31.1	3.36E-12	0.99976
MED13L;MED13	12:q24.21;17:q23.2	3.62E-12	0.99986
C20orf111	20:q13.12	3.72E-12	0.99988
SNX18;SNX9;SNX33	5:q11.2;6:q25.3;15:q24.2	3.77E-12	0.99988
E2F5;E2F6;E2F4	8:q21.2;2:p25.1;16:q22.1	4E-12	0.99993
NCOR2;NCOR1;C20orf191;ENSG00000205203	12:q24.31;17:p11.2;20:p11.1;17:p11.2	4.56E-12	0.99997
TEAD1;TEAD2;TEAD3;TEAD4	11:p15.2;19:q13.33;6:p21.31;12:p13.33	4.82E-12	0.99997
VASP;EVL;ENAH	19:q13.32;14:q32.2;1:q42.12	4.93E-12	0.99997
FAT3;FAT2;FAT1	11:q14.3;5:q33.1;4:q35.2	5.84E-12	0.99999
MCART6;MCART2;MCART1;ENSG00000189332	X:q22.2;18:q12.1;9:p13.2;11:p15.1	6.01E-12	1
MLXIPL;MLX;MLXIP	7:q11.23;17:q21.31;12:q24.31	6.5E-12	1
MKLN1	7:q32.3	6.72E-12	1
SYT1;SYT5;SYT2	12:q21.2;19:q13.42;1:q32.1	7.02E-12	1
TANK	2:q24.2	7.15E-12	1
ENSG00000188856	8:q21.13	8.52E-12	1
CADPS2;CADPS	7:q31.32;3:p14.2	8.57E-12	1
KRT8P9;KRT79;KRT7	12:q13.13;12:q13.13;12:q13.13	8.98E-12	1
CCDC11;TCHP	18:q21.1;12:q24.11	1E-11	1
TPR;PCNT;EEA1;AKAP9	1:q31.1;21:q22.3;12:q22;7:q21.2	1.03E-11	1
JMJD2A;JARID2;JMJD2D;JMJD2C;JMJD2B	1:p34.2;6:p23;11:q21;9:p24.1;19:p13.3	1.03E-11	1
SEC16A;SEC16B	9:q34.3;1:q25.2	1.15E-11	1

ENSA;ENSG00000128989;ENSG00000204637;ENSG00000177261	1:q21.2;15:q21.2;5:q35.3;2:q35	1.48E-11	1
RPL34	4:q25	1.5E-11	1
CLK1;CLK3;CLK2;CLK4	2:q33.1;15:q24.1;1:q22;5:q35.3	1.54E-11	1
SALL1;SALL4;SALL3	16:q12.1;20:q13.2;18:q23	1.58E-11	1
KRT14;KRT23;KRT20;KRT9;KRT16	17:q21.2;17:q21.2;17:q21.2;17:q21.2;17:q21.2	1.62E-11	1
TPX2	20:q11.21	1.73E-11	1
RPS17P5;RPS17P2;RPS17;ENSG00000182774	6:p12.3;5:q23.1;15:q25.2;15:q25.2	1.75E-11	1
SPOP;KBTBD4;SPOPL	17:q21.33;11:p11.2;2:q22.1	1.76E-11	1
ATAD2B;ATAD1;ATAD2	2:p23.3;10:q23.2;8:q24.13	1.79E-11	1
SESTD1	2:q31.2	1.91E-11	1
MEST	7:q32.2	1.95E-11	1
SRBD1	2:p21	2.24E-11	1
ATP11A;ATP11B;ATP11C	13:q34;3:q26.33;X:q27.1	2.27E-11	1
RAB13;RAB1A;RAB1B	1:q21.3;2:p14;11:q13.1	2.55E-11	1
UBP1;TFCP2L1;TFCP2	3:p22.3;2:q14.2;12:q13.13	2.55E-11	1
WIPI1;WDR45;WDR45L;WIPI2	17:q24.2;X:p11.23;17:q25.3;7:p22.1	2.6E-11	1
RBMS3;RBMS2P;RBMS1	3:p24.1;12:q13.3;2:q24.2	2.65E-11	1
CDC6;ORC1L	17:q21.2;1:p32.3	2.71E-11	1
MRPS35	12:p11.22	2.99E-11	1
SLC2A4RG;ZNF395;ZNF704	20:q13.33;8:p21.1;8:q21.13	3.01E-11	1
CCDC57	17:q25.3	3.07E-11	1
SNX19;SNX14;SNX25;SNX13	11:q24.3;6:q14.3;4:q35.1;7:p21.1	3.08E-11	1
CDK2;CDC2;CDK3	12:q13.2;10:q21.2;17:q25.1	3.13E-11	1
SLC38A1;SLC38A11	12:q13.11;2:q24.3	3.15E-11	1
CDY1;PECI;CDYL2;CDYL;CDY2A;CDY1B;ENSG00000182415	Y:q11.23;6:p25.2;16:q23.2;6:p25.1;Y:q11.221;Y:q11.223;Y:q11.221	3.25E-11	1
EIF4A2;EIF4A3;EIF4A1	3:q27.3;17:q25.3;17:p13.1	3.64E-11	1
HGSNAT	8:p11.21	3.64E-11	1
FBXL3;FBXL21	13:q22.3;5:q31.1	3.73E-11	1
RPS6KB2;RPS6KB1	11:q13.1;17:q23.1	3.74E-11	1
RPL22L1;RPL22P11	3:q26.2;1:p36.31	3.77E-11	1
NPAL2;NIPA1;NPAL3	8:q22.2;15:q11.2;1:p36.11	3.89E-11	1
REEP2;REEP3;REEP1	5:q31.2;10:q21.3;2:p11.2	3.94E-11	1
CBX1;CBX3;CBX5	17:q21.32;7:p15.2;12:q13.13	4.14E-11	1
LRBA;NBEAL1;NBEA;ENSG00000160796	4:q31.3;2:q33.2;13:q13.2;3:p21.31	4.24E-11	1
EBPL;EBP	13:q14.3;X:p11.23	4.35E-11	1

Query 3: Results for the GWAS query corresponding to **Fig. 1D**

Target	Band	Score	p-value
TERT	5:p15.33	1.35E-85	0
ENSG00000203587	5:p15.33	1.03E-83	0
ENSG00000196735	6:p21.32	3.25E-77	0
CLPTM1L	5:p15.33	3.49E-75	0
ENSG00000198599	6:p21.32	1.34E-69	0
ENSG00000196126	6:p21.32	3.85E-68	0
HLA-DQB1	6:p21.32	6.07E-68	0
SLC6A18	5:p15.33	3.21E-67	0
ENSG00000217141	6:p21.32	1.46E-64	0
ENSG00000215245	5:p15.33	3.59E-63	0
ENSG00000215221	9:p21.3	2.18E-61	0
SLC6A19	5:p15.33	5.61E-61	0
HLA-DRB5	6:p21.32	3.13E-59	0
ENSG00000196301	6:p21.32	1.34E-58	0
SLC6A3	5:p15.33	4.15E-58	0
CDKN2B	9:p21.3	1.03E-57	0
ENSG00000218589	6:p21.32	2.38E-57	0
CDKN2A	9:p21.3	7.73E-57	0
ENSG00000214861	6:p21.32	1.84E-56	0

HLA-DRA	6:p21.32	3.25E-56	0
BTNL2	6:p21.32	6.25E-56	0
SLC12A7	5:p15.33	1.62E-54	0
ENSG00000204276	6:p21.32	3.52E-54	0
LPCAT1	5:p15.33	1.73E-53	0
C6orf10	6:p21.32	1.47E-52	0
ENSG00000204275	6:p21.32	3.28E-52	0
NKD2	5:p15.33	6.44E-52	0
ENSG00000217297	9:p21.3	1.32E-51	0
MTAP	9:p21.3	3.59E-50	0
ENSG00000221244	5:p15.33	7.73E-50	0
ENSG00000185986	5:p15.33	6.24E-49	0
HLA-DOB	6:p21.32	6.78E-49	0
ENSG00000223335	6:p21.32	1.34E-48	0
ENSG00000215246	5:p15.33	1.62E-48	0
ENSG00000203594	5:p15.33	1.88E-47	0
ENSG00000188002	5:p15.33	2.36E-47	0
ENSG00000220534	6:p21.32	1.34E-46	0
HLA-C	6:p21.33	1.35E-46	0
TAP2	6:p21.32	5.2E-46	0
TRIP13	5:p15.33	1.24E-45	0
ENSG00000210529	9:p21.3	1.78E-45	0
ENSG00000204301	6:p21.32	3.66E-45	0
BRD9	5:p15.33	6.17E-45	0
ZDHHC11	5:p15.33	3.28E-44	0
ENSG00000204264	6:p21.32	4.65E-44	0
POU5F1P1	8:q24.21	3.79E-43	0
MYC	8:q24.21	6.61E-43	0
ENSG00000204261	6:p21.32	7.89E-43	0
ENSG00000220090	9:p21.3	1.21E-42	0
ENSG00000213654	6:p21.32	4.42E-42	0
ENSG00000168394	6:p21.32	1.72E-41	0
PBX2	6:p21.32	4.01E-41	0
ENSG00000218552	6:p21.33	2.69E-40	0
AGER	6:p21.32	7.13E-40	0
ENSG00000204259	6:p21.32	1.14E-39	0
IRF4	6:p25.3	1.51E-39	0
ENSG00000212988	8:q24.21	1.63E-39	0
ENSG00000214789	8:q24.21	1.64E-39	0
ENSG00000221315	8:q24.21	3.08E-39	0
HLA-Z	6:p21.32	4E-39	0
ENSG00000217440	9:p21.3	7.74E-39	0
RNF5	6:p21.32	5.87E-38	0
ENSG00000201658	6:p21.33	6E-38	0
ENSG00000204520	6:p21.33	7.5E-38	0
ENSG00000212066	6:p21.32	1.09E-37	0
HCP5	6:p21.33	1.5E-37	0
ENSG00000168384	6:p21.32	4.25E-37	0
ENSG00000204310	6:p21.32	6.57E-37	0
HLA-DMB	6:p21.32	1.22E-36	0
ENSG00000219108	6:p21.33	1.86E-36	0
ENSG00000216565	6:p21.33	2.15E-36	0
HLA-DPB1	6:p21.32	4.37E-36	0
HLA-DMA	6:p21.32	5.26E-36	0
ENSG00000217524	6:p21.33	8.59E-36	0
ENSG00000219359	6:p21.33	1.85E-35	0
ENSG00000221988	6:p21.32	3.84E-35	0
ENSG00000199332	6:p21.33	5.67E-35	0
ENSG00000214892	6:p21.33	1.01E-34	0
ENSG00000201680	6:p21.33	1.21E-34	0
ENSG00000172899	6:p21.32	2.22E-34	0
BRD2	6:p21.32	3.15E-34	0
ENSG00000217679	6:p21.33	3.34E-34	0
RPL3P2	6:p21.33	3.35E-34	0
ENSG00000204542	6:p21.33	4.22E-34	0
HLA-DOA	6:p21.32	5.53E-34	0

ENSG00000168452	6:p21.32	8.21E-34	0
ENSG00000204538	6:p21.33	8.98E-34	0
ENSG00000219309	6:p21.32	9.63E-34	0
ENSG00000216590	6:p21.33	1.56E-33	0
PRRT1	6:p21.32	1.95E-33	0
ENSG00000204516	6:p21.33	2.07E-33	0
ENSG00000204536	6:p21.33	4.37E-33	0
ENSG00000204539	6:p21.33	6.31E-33	0
ENSG00000222895	6:p21.33	8.26E-33	0
ENSG00000219797	6:p21.33	9.31E-33	0
ENSG00000204540	6:p21.33	9.62E-33	0
HCG27	6:p21.33	1.1E-32	0
DUSP22	6:p25.3	2.27E-32	0
COL11A2P	6:p21.32	2.59E-32	0
TCF19	6:p21.33	5.46E-32	0
